# Glycine/alginate-based piezoelectric film consisting of a single, monolithic β-glycine spherulite towards flexible and biodegradable force sensor

**DOI:** 10.1093/rb/rbae047

**Published:** 2024-05-11

**Authors:** Qiaoxia Lin, Yonggang Zhang, Luhua Chen, Haoyue Zhang, Chuanfeng An, Chengze Li, Qifan Wang, Jinhui Song, Wei He, Huanan Wang

**Affiliations:** MOE Key Laboratory of Bio-Intelligent Manufacturing, Dalian Key Laboratory of Artificial Organ and Regenerative Medicine, School of Bioengineering, Dalian University of Technology, Dalian 116024, Liaoning, P. R. China; State Key Laboratory of Fine Chemicals, Frontiers Science Center for Smart Materials Oriented Chemical Engineering, Dalian University of Technology, Dalian 116024, Liaoning, P. R. China; School of Chemical Engineering, Dalian University of Technology, Dalian 116024, Liaoning, P. R. China; MOE Key Laboratory of Bio-Intelligent Manufacturing, Dalian Key Laboratory of Artificial Organ and Regenerative Medicine, School of Bioengineering, Dalian University of Technology, Dalian 116024, Liaoning, P. R. China; School of Mechanical Engineering, Dalian University of Technology, Dalian 116024, Liaoning, P. R. China; MOE Key Laboratory of Bio-Intelligent Manufacturing, Dalian Key Laboratory of Artificial Organ and Regenerative Medicine, School of Bioengineering, Dalian University of Technology, Dalian 116024, Liaoning, P. R. China; State Key Laboratory of Fine Chemicals, Frontiers Science Center for Smart Materials Oriented Chemical Engineering, Dalian University of Technology, Dalian 116024, Liaoning, P. R. China; School of Chemical Engineering, Dalian University of Technology, Dalian 116024, Liaoning, P. R. China; MOE Key Laboratory of Bio-Intelligent Manufacturing, Dalian Key Laboratory of Artificial Organ and Regenerative Medicine, School of Bioengineering, Dalian University of Technology, Dalian 116024, Liaoning, P. R. China; MOE Key Laboratory of Bio-Intelligent Manufacturing, Dalian Key Laboratory of Artificial Organ and Regenerative Medicine, School of Bioengineering, Dalian University of Technology, Dalian 116024, Liaoning, P. R. China; MOE Key Laboratory of Bio-Intelligent Manufacturing, Dalian Key Laboratory of Artificial Organ and Regenerative Medicine, School of Bioengineering, Dalian University of Technology, Dalian 116024, Liaoning, P. R. China; School of Mechanical Engineering, Dalian University of Technology, Dalian 116024, Liaoning, P. R. China; State Key Laboratory of Fine Chemicals, Frontiers Science Center for Smart Materials Oriented Chemical Engineering, Dalian University of Technology, Dalian 116024, Liaoning, P. R. China; School of Chemical Engineering, Dalian University of Technology, Dalian 116024, Liaoning, P. R. China; MOE Key Laboratory of Bio-Intelligent Manufacturing, Dalian Key Laboratory of Artificial Organ and Regenerative Medicine, School of Bioengineering, Dalian University of Technology, Dalian 116024, Liaoning, P. R. China; State Key Laboratory of Fine Chemicals, Frontiers Science Center for Smart Materials Oriented Chemical Engineering, Dalian University of Technology, Dalian 116024, Liaoning, P. R. China

**Keywords:** piezoelectric biomaterials, glycine spherulite, flexible electronics, biodegradable sensor, composite

## Abstract

Development of piezoelectric biomaterials with high piezoelectric performance, while possessing excellent flexibility, biocompatibility, and biodegradability still remains a great challenge. Herein, a flexible, biocompatible and biodegradable piezoelectric β-glycine–alginate–glycerol (Gly-Alg-Glycerol) film with excellent *in vitro* and *in vivo* sensing performance was developed. Remarkably, a single, monolithic β-glycine spherulite, instead of more commonly observed multiple spherulites, was formed in alginate matrix, thereby resulting in outstanding piezoelectric property, including high piezoelectric constant (7.2 pC/N) and high piezoelectric sensitivity (1.97 mV/kPa). The Gly-Alg-Glycerol film exhibited superior flexibility, enabling complex shape-shifting, e.g. origami pigeon, 40% tensile strain, and repeated bending and folding deformation without fracture. *In vitro*, the flexible Gly-Alg-Glycerol film sensor could detect subtle pulse signal, sound wave and recognize shear stress applied from different directions. In addition, we have demonstrated that the Gly-Alg-Glycerol film sensor sealed by polylactic acid and beeswax could serve as an *in vivo* sensor to monitor physiological pressure signals such as heartbeat, respiration and muscle movement. Finally, the Gly-Alg-Glycerol film possessed good biocompatibility, supporting the attachment and proliferation of rat mesenchymal stromal cells, and biodegradability, thereby showing great potential as biodegradable piezoelectric biomaterials for biomedical sensing applications.

## Graphical Abstract

**Figure:**
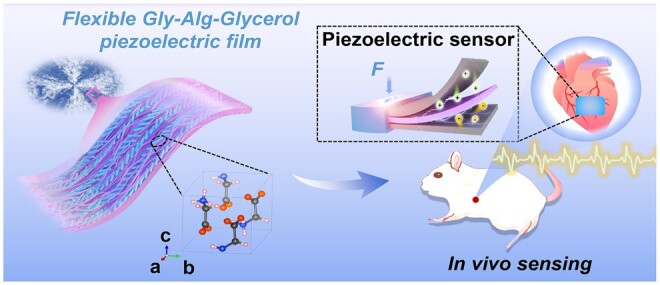

## Introduction

Piezoelectric effect is a phenomenon that describes ‘pressure-induced electricity’ for piezoelectric materials [1], where a linear and reversible transformation can be permitted between mechanical energy and electrical energy [2, 3]. So far, piezoelectric materials have been widely used in sensors [4, 5], actuators, transducers, catalysis [2, 6], and have become indispensable for modern life. Over the past few decades, the piezoelectric behavior has been found in a variety of biological tissues, including bone, skin, hair, tendon, cartilage, etc. [7]. Recently, with the emergence of biomedical flexible electronics for personalized healthcare, piezoelectric biomaterials with sensitive pressure responses, self-powered characteristics and simple engineered structures, have shown great potential for application in the fields of human physiological signal detection [3], self-powered bioelectronic device [8] and drug delivery [9]. To date, various piezoelectric materials have been explored for biomedical applications, such as zirconate titanate (PZT), zinc oxide (ZnO), barium titanate (BaTiO_3_) and poly(vinylidene fluoride) (PVDF) [1, 10]. These piezoelectric materials possess strong piezoelectric properties, which, however, have been criticized for their poor biocompatibility and even cytotoxicity. In addition, they are hardly biodegradable and are very fragile especially for those inorganic piezoelectric materials. Some of them are even extremely hazardous to the natural environment. All these disadvantages have hindered their practical applications in biomedical field [11]. To address these issues, a few attempts have been made to develop flexible, biocompatible, biodegradable and environmentally friendly piezoelectric materials by using synthetic biopolymers, e.g. poly-l-lactic acid (PLLA), to achieve strong piezoelectric responses, while the fabrication techniques are quite complex [12, 13]. Therefore, there is a tremendous need for the development of novel piezoelectric biomaterials with easy accessibility, flexibility, biodegradability and biocompatibility.

Piezoelectric biomaterials, including native biomaterials such as spider silk [14], eggshell membrane [15] and onion skin [16], as well as assembled products of amino acids [17], diphenylalanine (FF) peptide nanotubes [18, 19], collagen [20], cellulose [21], chitin [22] and bacteriophage [23], are regarded as the most potential alternatives to traditional piezoelectric materials due to their accessibility, biocompatibility and biodegradability [24]. However, the weak piezoelectric effect caused by the random polarization and the absence of large-scale domain orientation alignment have greatly limited their application [25]. Among these piezoelectric biomaterials, glycine (Gly), the simplest achiral amino acid, has received great attention due to its extremely high piezoelectric constant in beta (β)-phase [17] and is currently considered as the most promising piezoelectric biomaterial [26].

Gly mainly exists in three polymorphic forms, namely alpha (α), beta (β) and gamma (γ) phases [27]. α-Gly is in centrosymmetric space group P2_1_/n without piezoelectric property [28]. On the contrary, β-Gly and γ-Gly belonging to non-centrosymmetric space groups P2_1_ and P3_2_, are piezoelectric [29, 30]. β-Gly has a much higher piezoelectric coefficient (*d*_16_ = 178 pC/N) than that of γ-Gly (*d*_33_ = 10 pC/N) [17]. However, β-Gly is unstable under ambient conditions which tends to transform into α- or γ-Gly phase [31], therefore is difficult to obtain and has been rarely reported. Hosseini *et al*. developed a flexible piezoelectric film consisting of pure β-Gly spherulite embedded in a chitosan matrix through a simple solvent-casting method. The β-Gly/chitosan film was slightly flexible with piezoelectric sensitivity comparable to those of nondegradable commercial piezoelectric materials. However, its *in vivo* biocompatibility and biodegradability was unknown. In addition, the β-Gly/chitosan film was composed of multiple spherulite domains [11], which has been previously revealed to have different polarization orientations and, in turn, partially canceled out, resulting in weak macroscopic piezoelectricity [32]. Recently, pure β-Gly film with aligned domains across the entire film was developed by Zhang *et al*. via synergistic nanoconfinement and *in situ* poling. The β-Gly film exhibited an enhanced piezoelectric strain coefficient and an exceptional piezoelectric voltage coefficient, as well as excellent output performance and thermostability. Nevertheless, the method fails to fabricate β-Gly films that are both flexible and free-standing [33]. All in all, it still remains a great challenge to develop flexible, biocompatible and biodegradable free-standing β-Gly film with high piezoelectric performance.

**Scheme 1.**
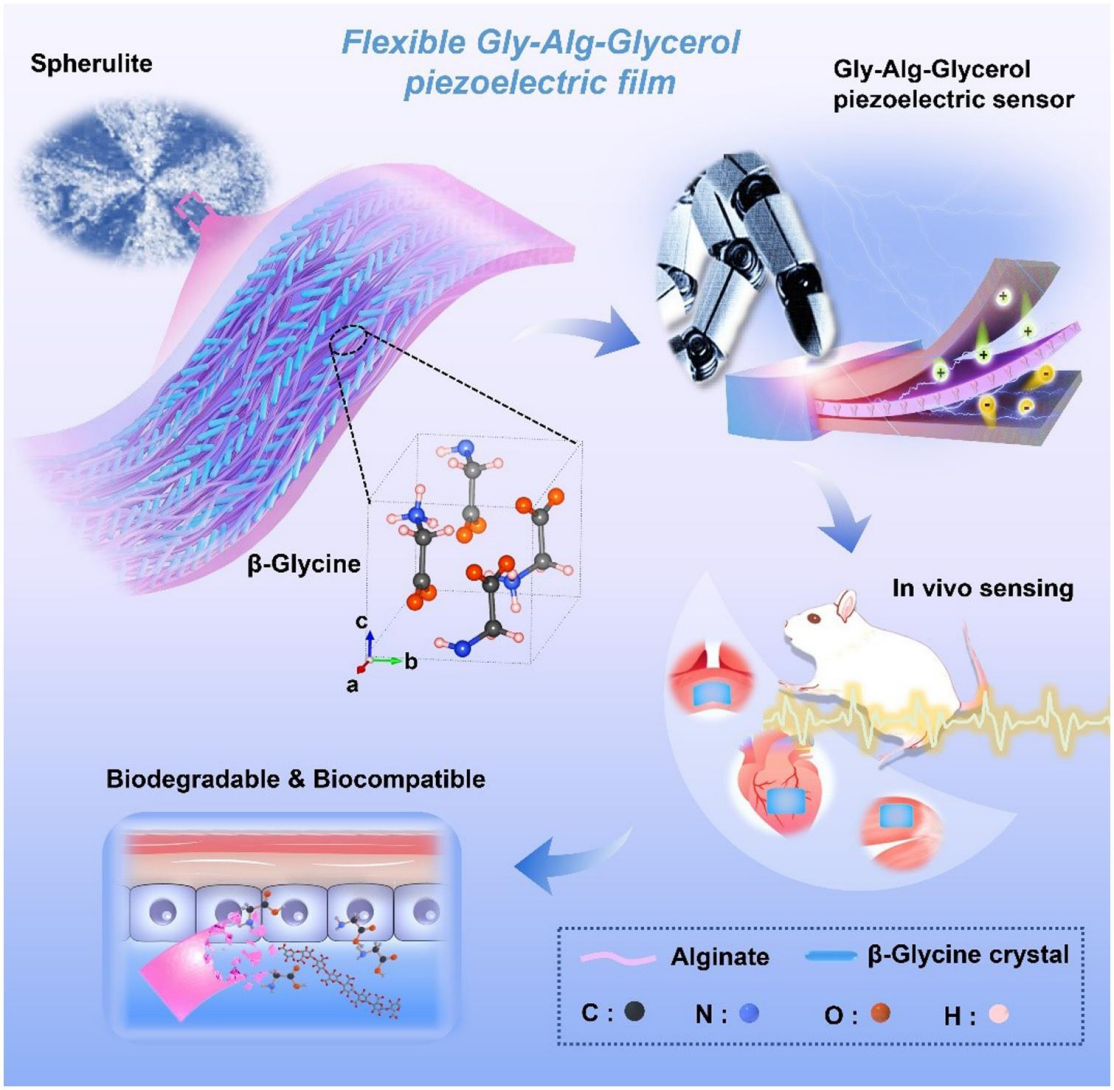
Schematic illustration of the flexible and biodegradable Gly-Alg-Glycerol piezoelectric film production and its biomedical application as piezoelectric sensor.

Herein, we fabricated piezoelectric films consisting of pure β-Gly crystals embedded in alginate (Alg) and glycerol matrix by a simple solvent-casting method (Scheme 1). Be different from previous studies, the β-Gly crystals formed a single, monolithic spherulite, rather than multiple spherulites, thus endowing the piezoelectric films with the excellent piezoelectric properties. The piezoelectric sensor prepared using Gly-Alg-Glycerol films could monitor weak physiological pressure signals such as rat heartbeat, respiration, muscle contraction and human pulse. Moreover, the Gly-Alg-Glycerol piezoelectric films showed outstanding flexibility, which could be easily processed into complex shapes, e.g. origami pigeon. *In vitro* tests revealed that the Gly-Alg-Glycerol piezoelectric films supported mesenchymal stromal cells (MSCs) proliferation. Additionally, the Gly-Alg-Glycerol piezoelectric films were fully degradable *in vivo*. Taken together, this easily accessible, flexible, biocompatible and biodegradable piezoelectric film presents an appealing alternative to traditional piezoelectric materials for biomedical applications.

## Materials and methods

### Materials

Sodium alginate was purchased from Macklin (China). Glycine, Triton X-100, bovine serum albumin, Cell Counting Kit-8 and 4% paraformaldehyde tissue fixative were purchased from Solarbio (China). Dichloromethane, chitosan, beeswax and glycerol were purchased from Aladdin (China). Polylactic acid was purchased from Shenzhen Esun Industrial Co., Ltd (China). Paraformaldehyde was purchased from Sigma-Aldrich (USA). Tissue adhesive was purchased from 3M (USA). PDMS (RTV-615) was purchased from Momentive (USA). Kapton tape was purchased from DuPont (USA). Minimal essential medium α (α-MEM), fetal bovine serum, penicillin-streptomycin, phosphate-buffered saline (PBS) and trypsin were purchased from Gibco (USA). The calcein-AM and propidium iodide double staining kit, 4′,6-diamidino-2-phenylindole and TRITC phalloidin were purchased from Invitrogen (USA). Sprague–Dawley (SD) rats were obtained from Shanghai Slake Experimental Animal Co., Ltd (China). All reagents were used as received without further purification.

### Fabrication of Gly-Alg-Glycerol film

Fabrication of Gly-Alg-Glycerol films with a Gly/Alg mass ratio of 1:1 and different amount (0.5%, 1%, 2% and 3% (w/v)) of glycerol: First, a Gly-Alg solution (1:1) was prepared by dissolving 0.4 g sodium alginate and 0.4 g Gly in 40 ml of deionized water. Then, 0.2, 0.4, 0.8 and 1.2 g glycerol were added into the above Gly-Alg solution, respectively. Finally, 40 ml of the mixed solutions was casted into a 115-mm-diameter polystyrene petri dish and dried at room temperature for 1 week.

Fabrication of Gly-Alg-Glycerol films with 1% (w/v) glycerol and different Gly/Alg mass ratios of 0.5:1, 1:1, 1.5:1 and 2:1: First, an Alg-Glycerol solution containing 1% w/v Alg and 1% w/v glycerol was prepared by mixing 0.4 g glycerol and 0.4 g sodium alginate with 40 ml of deionized water. Then, 0.2, 0.4, 0.6 and 0.8 g Gly powder were added into the above Alg-Glycerol solution, respectively. Finally, 40 ml of the mixed solutions were casted into a 115-mm-diameter polystyrene petri dish and dried at room temperature for 1 week.

### Fabrication of encapsulated Gly-Alg-Glycerol piezoelectric sensor

In a typical procedure, a layer of magnesium with a thickness of 100 nm was deposited onto both sides of the Gly-Alg-Glycerol piezoelectric film as electrodes by vacuum evaporation. Two conductive wires covered with insulation layer were connected to the upper and lower electrodes. Then, the Gly-Alg-Glycerol film together with the electrodes and the conductive wires were coated with a layer of beeswax by dip-coating in molten beeswax at 80°C. A PLA film with a thickness of 10 μm was prepared by spin-coating of dichloromethane solution containing 10% (w/v) PLA on a silicon wafer at a speed of 500–1000 r/min. Finally, the sensor was further placed between two layers of PLA film and then sealed by hot pressing to enhance its waterproof ability. The thickness of the encapsulated piezoelectric sensor is about 90–100 μm.

### Characterization of Gly-Alg-Glycerol film

#### Structural analysis and characterization

The crystallization process of the Gly-Alg-Glycerol film were observed using optical microscope (Nikon, ECLIPSE E100, Japan). The orientation of β-Gly crystals was characterized using cross-polarized optical microscope (Nikon, ECLIPSE LV100N POL, Japan). The morphology of the β-Gly crystals embedded in the Gly-Alg-Glycerol film was observed using field-emission scanning electron microscopy (FEI, NOVA NanoSEM 450, USA). XRD analysis was performed using X-ray powder diffraction (Rigaku, MiniFlex 600, Japan, Cu Kα radiation, *λ* = 1.54059 Å) in the range of 5°−80° in increments of 0.01°. The thickness of the Gly-Alg-Glycerol film was measured using profilometer (Bruker, DektakXT stylus profiler, Germany).

#### Mechanical test

The tensile tests of Gly-Alg-Glycerol films with dimensions of 10 × 30 × 0.05 mm^3^ were tested at a strain rate of 5 mm/min on a universal testing machine (E43, MTS instrument, USA), with a 50 N load cell.

#### Piezoelectric and dielectric properties characterization

The piezoelectric properties of Gly-Alg-Glycerol film under two modes of mechanical stimuli, compression and bending, were evaluated by measuring the open-circuit output voltage using a homemade piezoelectric testing system. The Gly-Alg-Glycerol film was sandwiched between two Ag electrodes and packaged with Kapton tape for testing. The open-circuit output voltage was recorded by a digital multimeter (Keithley, DMM 6500, USA).

In the compression mode, the piezoelectric sensor was fixed on a micro-displacement adjusting platform; whereas its top surface was subjected to cyclic compression applied by an electromechanical shaker. In addition, a force sensor was placed between the piezoelectric sensor and the micro-displacement adjusting platform. The compression force was regulated by the micro-displacement adjusting platform and recorded by the force sensor integrated with an automated high-speed measurement system. The corresponding output voltage signal was recorded by the digital multimeter. In the bending mode, the piezoelectric sensor was attached to a 5-mm-thick PDMS matrix. The PDMS matrix was then fixed and bent cyclically by a vibration push rod. The output voltage signal was recorded by the digital multimeter. All tests were performed under ambient conditions (∼25°C and ∼45% relative humidity).

The *d*_33_ piezoelectric coefficient of the Gly-Alg-Glycerol films was measured by a quasistatic d_33_ piezometer (Institute of Acoustics, Chinese Academy of Sciences, ZJ-3AN, China). The relative permittivity is examined by a precision impedance analyzer (Agilent Technologies, 4294A, USA).

### Biocompatibility evaluation of Gly-Alg-Glycerol film

#### Cell toxicity

A series of culture medium solutions with different concentrations (0, 0.01, 0.02, 0.05, 0.1, 0.2, 0.5 and 1 mg/ml) of Gly-Alg-Glycerol were prepared by dissolving the sterilized Gly-Alg-Glycerol film in minimal essential medium (α-MEM). The films were sterilized by irradiation with Co-60 gamma rays. Primary bone marrow-derived MSCs were isolated from 3-week-old male SD rats according to the previous report [34]. The experiment was carried out in accordance with the regulation of Biomedical and Animal Ethics Committee of Dalian University of Technology and approved with DUTSBE230517-01. The cells were seeded in a 24-well plate at a density of 5 × 10^3^/well and incubated for 24 h at 37°C and 5% CO_2_. Then the culture medium was refreshed with the culture medium solutions containing different concentrations of Gly-Alg-Glycerol. Following culture for 1, 2 and 3 days, the cells were washed with PBS. Subsequently, cell viability was detected using the CCK-8 kit and live/dead cell staining kit. To quantify the number of live cells, 1 ml CCK-8 solution (10% in basic cell culture medium) was added to each well. After incubation at 37°C for 2 h, the absorbance was measured on the microplate reader (Bio-Rad iMark, USA) at 450 nm. The relative cell viability was expressed as (*A*_test –_ *A*_blank_)/(*A*1_control –_ *A*_blank_) × 100%, where *A*1_control_ is the optical density of the wells with cells cultured in culture media without any film solutes at day 1. To visualize the distribution of live/dead cells, the cells were washed with PBS, followed by adding calcein-AM and propidium iodide dye solution. Then, the plate was incubated at 37 °C for 30 min before being rinsed with PBS three times. The staining images were captured using a confocal laser scanning microscope (Olympus FV3000, Japan).

#### Cell morphology

After 2 days of culture in the culture medium solutions containing different concentrations of Gly-Alg-Glycerol, the cytoskeleton and nucleus were stained with TRITC phalloidin (red) and 4′,6-diamidino-2-phenylindole (blue), respectively. In brief, the cells were fixed with 4% formaldehyde for 15 min and washed three times with PBS, followed by treatment with 0.1% (v/v) Triton X-100 for 15 min at room temperature. Subsequently, the cell samples were washed three times with PBS and blocked with 1 wt% bovine serum albumin. Finally, the samples were stained with TRITC phalloidin for 1 h and 4′,6-diamidino-2-phenylindole for 10 min at 37°C, respectively. After staining, the cells were observed with a confocal laser scanning microscope (Olympus FV3000, Japan).

#### Hemolysis test

The experiment was approved by the Biomedical and Animal Ethics Committee of Dalian University of Technology (DUTSBE240314-2). Healthy rabbit whole blood was collected in tubes and washed three times with standard saline solution. After 4% red blood cells suspension being prepared, different concentrations of Gly-Alg-Glycerol were incubated with it at 37°C. The red blood cells were also incubated with standard saline solution and distilled water as negative control and positive control, respectively. After 1 h, the mixtures were centrifuged for 5 min at 2500 r/min. The absorbance values at 545 nm of the supernatant were measured. The hemolysis ratio (%) was determined by the following equation,

$$\text{Hemolysis ratio}\;\left(\%\right)=\left(\left({\text{OD}}_{\text{sample}\;-\;}{\text{OD}}_{\text{negative}}\right)/\left({\text{OD}}_{\text{positive}\;-\;}{\text{OD}}_{\text{negative}}\right)\right)\;\times \;100\%.$$

#### In vivo biocompatibility test

The animal experiment was approved by the Biomedical and Animal Ethics Committee of Dalian University of Technology (DUTSBE230216-001). The Gly-Alg-Glycerol films were sterilized by irradiation with Co-60 gamma rays and then implanted into subcutaneous pockets made on the dorsal back of SD rats. At day 1, 2, 4 and 7, the rats were sacrificed and the target tissues were harvested. The tissues were fixed, dehydrated, embedded in paraffin, sliced and stained by H&E.

### *In vitro* and *in vivo* sensing

#### In vitro sensing

To detect the pulse signal of human carotid artery, a Gly-Alg-Glycerol sensor was attached to the surface of carotid artery. The real-time output voltage signal of the Gly-Alg-Glycerol sensor was recorded by a digital multimeter. To detect sound wave signal, the sensor was placed in front of a speaker. Then the speaker was turn on, and the output voltage signal of the Gly-Alg-Glycerol sensor was recorded by a digital multimeter. The stability of the encapsulated Gly-Alg-Glycerol sensor was tested by immersing the sensor in a PBS at 37°C for 6 days. Each day, the sensor was removed from the solution for piezoelectric performance testing and then placed back.

#### In vivo sensing

Adult male SD rats were anesthetized and fixed in a supine position. The hair on the chest, abdomen and leg were removed. For heartbeat sensing, the chest cavity of the rat was opened to expose the heart, and an encapsulated Gly-Alg-Glycerol sensor was attached to the outer wall of the heart by using tissue adhesive. The incision was then sutured. The two conductive wires of the implanted sensor came out of the incision and were connected to a digital multimeter. A respirator was used to serve as a life-sustaining device during the experiment. For muscle activity sensing, an incision was made on the hind leg of a rat, and the encapsulated Gly-Alg-Glycerol sensor was implanted into the surface of quadriceps muscle. For diaphragmatic contraction sensing, an incision was made on the abdomen of a rat, and the encapsulated Gly-Alg-Glycerol sensor was implanted into the base of the diaphragm. The incisions were then sutured while leaving the conductive wires of the sensors subjected to air connecting to a multimeter as described above. The dimension of all the packaged Gly-Alg-Glycerol sensors used is 5 mm × 5 mm. Upon finishing all the tests, all rats were euthanized.

### Statistical analysis

The data were taken from at least three independent experiments and all obtained data was presented as mean value ± standard deviation (SD). Statistical analysis was performed using GraphPad Prism 5. One-way analysis of variance was used to determine the statistical significance of the results. Differences were considered significant when *P* < 0.05.

## Results and discussion

### Fabrication of Gly-Alg-Glycerol film

**Figure 1.**
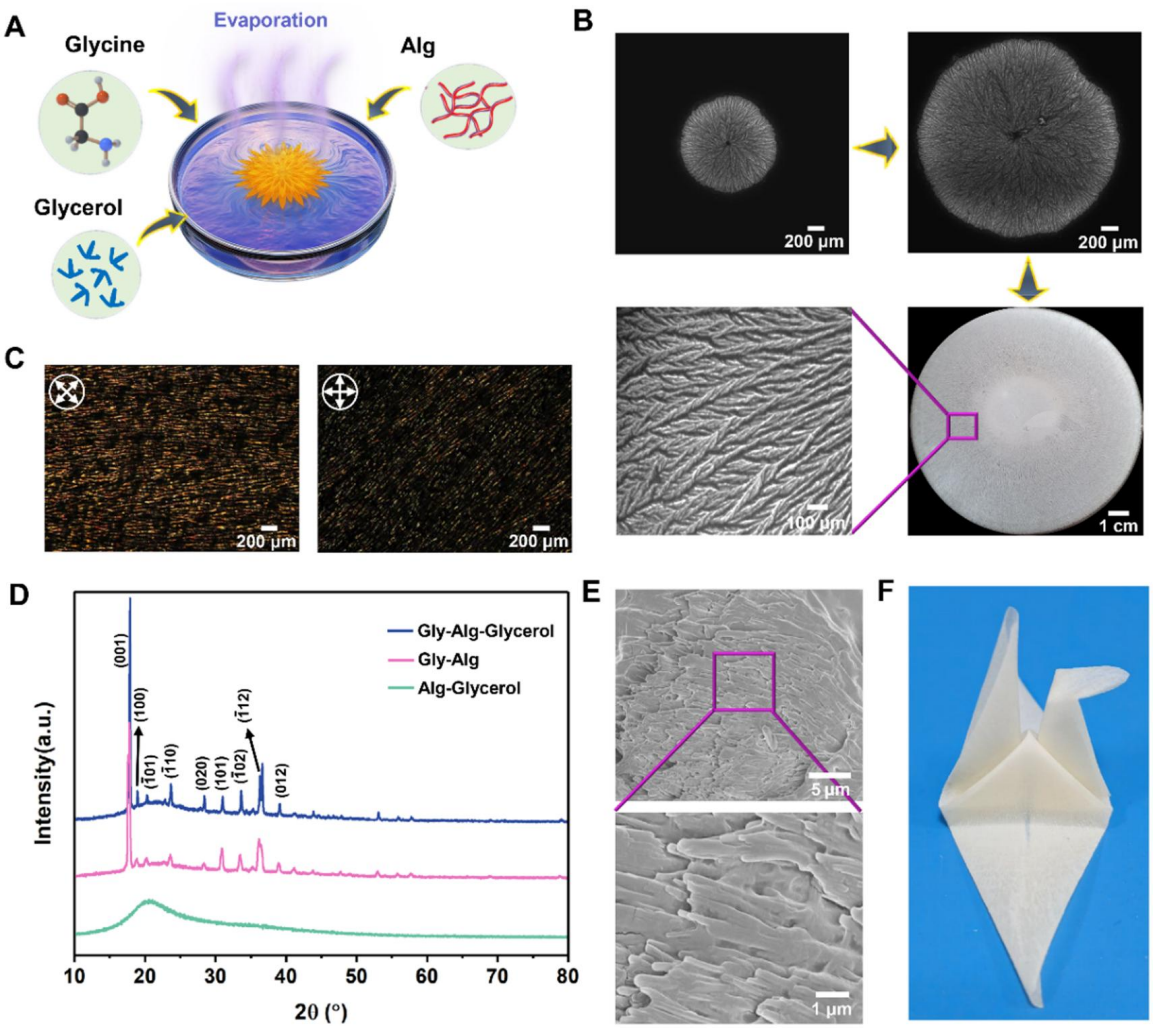
Synthesis and characterization of the Gly-Alg-Glycerol piezoelectric film. (**A**) Schematic illustration of the synthetic approach for the Gly-Alg-Glycerol piezoelectric films. (**B**) Optical microscopy images and digital photograph showing the crystallization process of a mixed aqueous solution containing 1% glycerol and Gly and Alg with a Gly/Alg ratio of 1:1. (**C**) POM images of the Gly-Alg-Glycerol film with 1% glycerol and a Gly/Alg ratio of 1:1 before and after rotation of 45°. The crossed arrows indicate the orientation of the crossed polarizers. (**D**) XRD spectra of the Gly-Alg-Glycerol film with 1% glycerol and a Gly/Alg ratio of 1:1, Alg-Glycerol film and Gly-Alg film prepared by the same method. (**E**) SEM images of the Gly-Alg-Glycerol film with 1% glycerol and a Gly/Alg ratio of 1:1. (**F**) a origami pigeon made of a piece of Gly-Alg-Glycerol film with 1% glycerol and a Gly/Alg ratio of 1:1.

The Gly-Alg-Glycerol piezoelectric films were fabricated by solvent-casting of mixed aqueous solution containing Gly, Alg and glycerol at room temperature (Figure 1A). The crystallization process of the Gly-Alg-Glycerol film was observed using microscope. As the water solution continues to evaporate, Gly initially nucleated near the center of the petri dish, followed by continuous growth, expanding rapidly across the entire dish. During the crystallization process, Gly crystals grew predominantly in the radial directions and formed a big spherulite with dendritic structure (Figure 1B). A polarized optical microscopy (POM) micrograph with a dark Maltese cross pattern shows the nucleation center and radial structure of the spherulite (Supplementary Figure S1). Interestingly, be different from previous studies, only one, monolithic β-Gly spherulite was formed throughout the entire film, rather than more commonly observed multiple spherulites (Supplementary Figure S2) [11], which, to the best of our knowledge, has not been reported elsewhere. Alg plays an important role in inducing the crystal types and monolithic spherulite arrangement of Gly. It is known that polymers can regulate the crystallization and crystal assembly of nonpolymeric materials [35]. When there are polymers in the solution, the primary nucleation of Gly is heterogeneous and being controlled by the polymer surface, upon which nucleation can occur faster due to the lowered nucleation potential barrier [36]. Different from chitosan polymers, containing a large number of amino groups, used to control Gly to form multiple spherulites in literature [11], Alg contains many negatively charged carboxylate functional groups generated by dissociation in aqueous solution. There are electrostatic and hydrogen bonding interactions between the polar groups (amino and carboxyl) of Gly and the functional groups of polymers, which affect the distribution and diffusion of Gly molecules in solution, thus controlling the nucleation, growth, and the subsequent assembly of Gly crystals. As is well known, the dissociation degree of carboxyl groups in Gly molecules (pI = 5.97) in aqueous solution is greater than that of amino groups. Therefore, we infer that the affinity between Alg polymer chains and Gly molecules is lower than that of chitosan, which is disadvantageous for the enrichment of Gly molecules around the Alg polymer chains, allowing Gly molecules to have higher diffusion energy, thereby hindering the formation of Gly spherulite nuclei and promoting the earliest formed nucleus to grow into a large spherulite. Figure 1C shows the POM images of an area of Gly-Alg-Glycerol film at different polarization angles, from which, when the film was rotated by 45°, majority of crystal regions extinguished the polarized light, suggesting Gly crystals in the Gly-Alg-Glycerol film exhibited a certain degree of anisotropy.

The X-ray diffraction (XRD) spectra of the Alg-Glycerol, Gly-Alg and Gly-Alg-Glycerol film are shown in Figure 1D. Alg-Glycerol film showed no diffraction peaks, which agrees with the amorphous nature of Alg and glycerol. The diffraction peaks of Gly-Alg film could be indexed to a single phase of β-Gly, suggesting the formation of pure β-Gly [37, 38] (Supplementary Figure S3). It has been shown that Alg played a significant role in regulating the crystallization of Gly molecules, since more thermodynamically stable α-Gly was formed instead of β-Gly when there was no Alg (Supplementary Figure S4). No obvious difference was observed between the XRD spectrum of the Gly-Alg and Gly-Alg-Glycerol film, suggesting that the presence of glycerol had limited effect on the crystallization of the Gly crystals. In previous studies, β-Gly was mainly synthesized by anti-solvent method or freeze-drying method. These β-Gly were unstable under ambient conditions which transformed into α- or γ-Gly phase rapidly [31, 39]. On the contrary, the β-Gly in the Gly-Alg-Glycerol piezoelectric film was very stable which did not show phase transition even after six months of storage. (Supplementary Figure S5). The Gly crystals embedded in the Alg-Glycerol matrix exhibited a needle-shape morphology (Figure 1E), consistent with the literature [39, 40]. We assumed that the confined space and the possible hydrogen bond interaction between the Gly molecules and Alg [41] endowed the β-Gly crystals with high stability.

### Mechanical properties of Gly-Alg-Glycerol film

The flexibility of piezoelectric films is crucial for their integrated application in flexible electronic systems. Gly crystals are rigid, making them rather challenging to be used in flexible sensing systems alone. An effective way to obtain flexibility is to embed Gly crystals in a flexible polymer matrix. Herein, we incorporated Alg into the Gly piezoelectric film, which not only regulated the formation and stability of the β-Gly crystals (Figure 1D), but also endowed the film with certain deformability. However, the Gly-Alg film was still too brittle to withstand large deformation (Movie S1). To further improve its flexibility, glycerol was introduced into the Gly-Alg film as a plasticizer. Glycerol is a low molecular weight hydrophilic molecule that is easy to incorporate into Alg polymer chains by forming hydrogen bonds with active polymer groups. It will weaken the intermolecular interactions inside the Alg polymer and increase the mobility of polymer chains, making the film more flexible [42, 43]. Due to the presence of glycerol, the Gly-Alg-Glycerol film allowed repeated bending and folding deformation without fracture (Movie S2). A piece of Gly-Alg-Glycerol film was folded into an origami pigeon (Figure 1F), demonstrating its outstanding flexibility.

**Figure 2.**
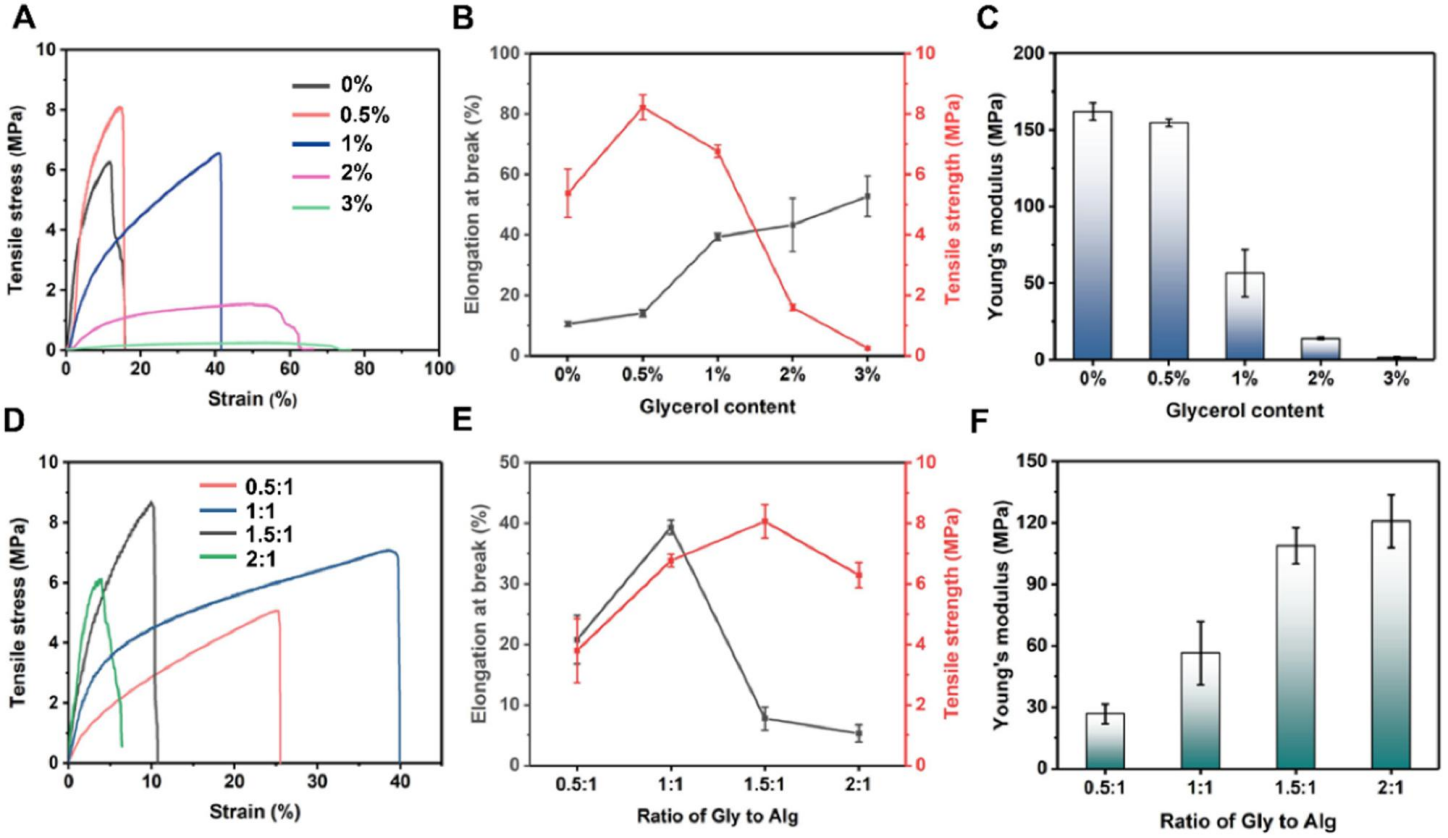
Mechanical properties of Gly-Alg-Glycerol film. (**A**) Tensile stress–strain curves, (**B**) elongation at break and tensile strength and (**C**) Young’s modulus of the Gly-Alg-Glycerol films with a Gly/Alg ratio of 1:1 and different amount of glycerol. (**D**) Tensile stress-strain curves, (**E**) elongation at the break and tensile strength and (**F**) Young’s modulus of the Gly-Alg-Glycerol films with 1% glycerol and different Gly/Alg mass ratios.

To further quantify the flexibility, the tensile mechanical behavior of the Gly-Alg-Glycerol film was studied using universal mechanical testing machine. We found that the content of glycerol had a significant impact on the flexibility of the piezoelectric film (Figure 2A), as reflected in elongation at break, tensile strength (Figure 2B) and Young’s modulus (Figure 2C). The elongation at break of the Gly-Alg-Glycerol film increased with the increase of the amount of glycerol, while both the tensile strength and Young’s modulus decreased as the content of the glycerol increased from 0.5% to 3%. When the amount of Glycerol was 0.5%, the improvement in flexibility was limited. However, when the amount of Glycerol was increased to 1%, the piezoelectric film became extremely flexible, with ultimate tensile strain up to 40%, about two times that of the piezoelectric film without glycerol. Based on the above results, we chose 1% glycerol for the subsequent experiments.

The influence of the Gly/Alg mass ratio on the mechanical properties of the piezoelectric films was further investigated. Figure 2D shows the tensile stress–strain curves of the Gly-Alg-Glycerol films with different Gly/Alg ratios. Apparently, when more Gly was added, the tensile strength and Young’s modulus of the piezoelectric films exhibited a remarkable uptrend (Figure 2E and F), whereas the tensile strength of the piezoelectric films with the Gly/Alg ratio of 2:1 decreased compared to that of the films with the Gly/Alg ratio of 1.5:1. The reason for that might be more Gly leading to the formation of more internal cavities in the films. Among all the samples, the film with the Gly/Alg ratio of 1:1 possessed the highest elongation at break, and relatively high tensile strength and Young’s modulus, indicating its superior mechanical flexibility, which means it can endure higher deformation and larger applied stress. Therefore, Gly-Alg-Glycerol films with 1% glycerol and a Gly/Alg ratio of 1:1 were used for the following experiments unless otherwise specified.

### Piezoelectric properties of Gly-Alg-Glycerol film

**Figure 3.**
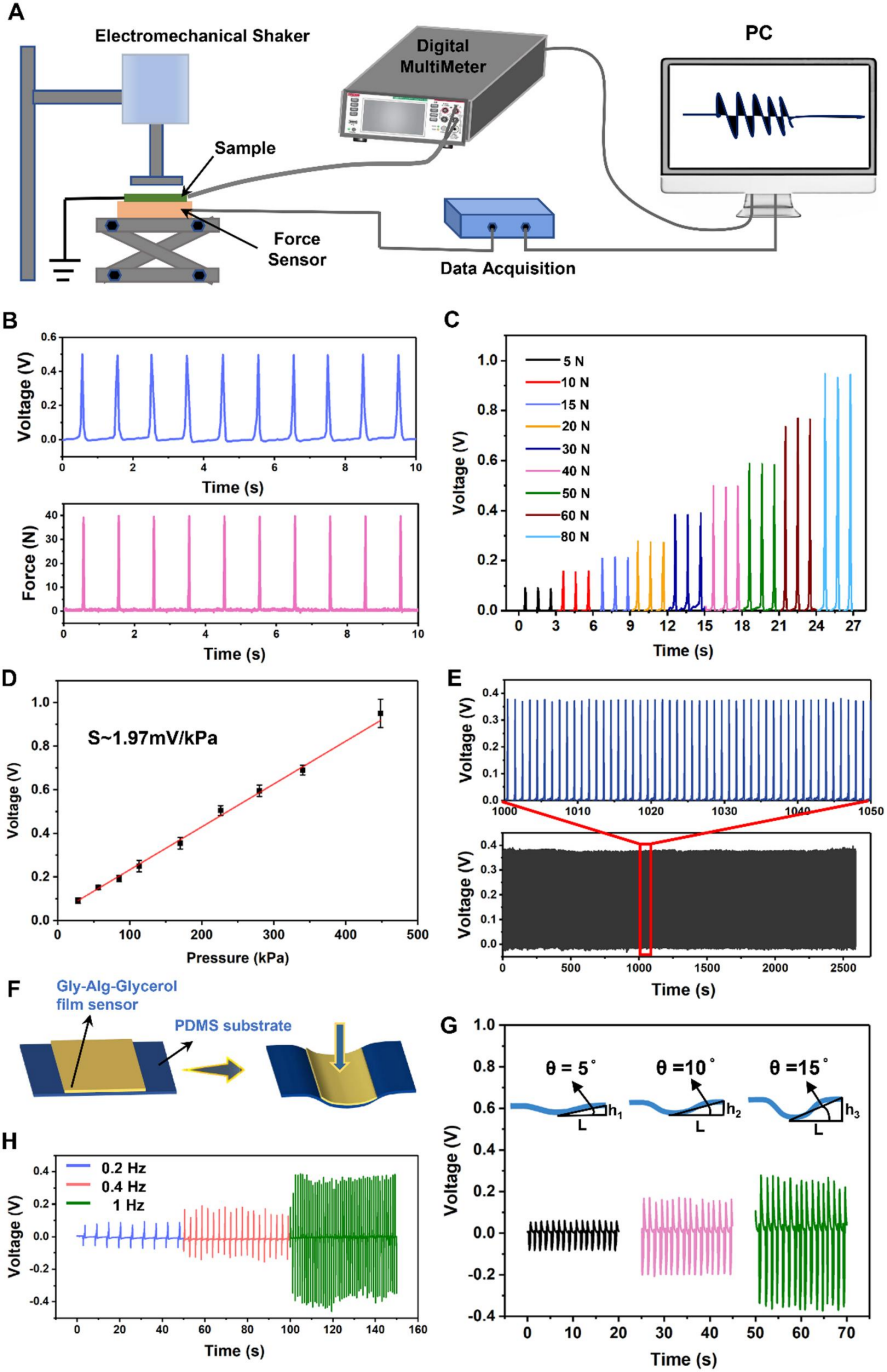
Piezoelectric property of Gly-Alg-Glycerol film. (**A**) Schematic illustration of the setup used to assess the piezoelectric performance of the Gly-Alg-Glycerol film. (**B**) Real-time output voltage generated by the Gly-Alg-Glycerol film in correlation to a cyclic impulse force of 40 N. (**C**) Output voltage generated by the Gly-Alg-Glycerol film subjected to a series of cyclic impulse force ranging from 5 to 80 N with a frequency of 1 Hz. (**D**) Voltage sensitivity of the piezoelectric film sensor under the pressure in the range of 28–448 kPa. (**E**) Long-term stability of the output voltage of the piezoelectric film tested under a cyclic impulse force of 30 N with a frequency of 1 Hz. (**F**) Schematic illustration of testing the piezoelectric performance of the Gly-Alg-Glycerol piezoelectric film under bending excitation. (**G**) Output voltage generated by the Gly-Alg-Glycerol film under cyclic bending at 5°, 10° and 15°, respectively, with a bending frequency of 0.8 Hz. (**H**) Real-time output voltage generated by the piezoelectric film under cyclic bending at 15° with different frequencies.

The macroscopic piezoelectricity of the Gly-Alg-Glycerol film was assessed using a homemade piezoelectric testing system (Figure 3A). Briefly, a piece of Gly-Alg-Glycerol film with a thickness of 50 μm was sandwiched between two Ag electrodes and then packaged with Kapton tape to serve as a piezoelectric sensor. A conductive shielding layer was attached to the sensor’s surface to prevent interference from triboelectric signals [44]. The sensor was then cyclically compressed and released by an electromechanical shaker. The applied force was controlled by a force sensor and a micro-displacement adjusting table, and the output voltage of the sensor was recorded by a digital multimeter. As shown in Figure 3B, when a 40 N impulse force was cyclically applied to the film over an area of 176.7 mm^2^ at a frequency of 1 Hz, the Gly-Alg-Glycerol film could generate a stable output voltage of 500 mV, which is significantly higher than the 210 mV of the multiple spherulites film under the same force stimulation (Supplementary Figure S2). The piezoelectric voltage of the Gly-Alg-Glycerol films varied with the content of glycerol and the Gly/Alg mass ratio. Gly-Alg films without glycerol exhibited the highest piezoelectric output voltage. The more glycerol, the lower output voltage of the piezoelectric film (Supplementary Figure S6). It can be explained that the presence of the glycerol increased the dielectric constant of the film, thereby reducing its piezoelectric output voltage (Supplementary Figure S7). It has been shown that a higher Gly/Alg ratio always resulted in a larger piezoelectric output voltage probably attributed to denser Gly or better domain alignment of Gly crystals (Supplementary Figure S8). Nevertheless, as mentioned earlier, when the Gly/Alg ratio was higher than 1:1, the Gly-Alg-Glycerol film became fragile, making them unsuitable for application in flexible electronic systems.

The piezoelectric sensitivity and piezoelectric stability of the Gly-Alg-Glycerol film were measured to prove its suitability for pressure sensing. To assess the piezoelectric sensitivity of the Gly-Alg-Glycerol film, we exposed the film to a series of cyclic impulse force ranging from 5 to 80 N with a frequency of 1 Hz while recording the corresponding output voltage. As shown in Figure 3C, the output voltage of the Gly-Alg-Glycerol film increased with the increase of the applied force. In addition, a good linear relationship was observed between the output voltage and the pressure in the range of 28–448 kPa, and the piezoelectric sensitivity of the film was up to 1.97 mV/kPa (Figure 3D). The d_33_ piezoelectric constant of the film quantified by a quasi-static *d*_33_ measuring instrument was as high as 7.2 ± 0.1 pC/N, which is higher than those of amino acid-based composites reported previously (Supplementary Figure S9) [25, 45]. The piezoelectric voltage coefficient g_33_ was calculated as 52 × 10^−3  ^Vm/N through the piezoelectric strain coefficients and the dielectric permittivity via the equation *g*_33_ = *d*_33_/*ε*_33_, in which *ε*_33_ = *ε*_r_ × *ε*_0_. We then performed a more than 2500-cycle test under cyclic impulse force of 30 N with a frequency of 1 Hz to evaluate the piezoelectric stability of the Gly-Alg-Glycerol film. During the test, the Gly-Alg-Glycerol film could produce very stable output voltage, indicating its excellent stability (Figure 3E).

The piezoelectric performance of the Gly-Alg-Glycerol film under cyclic bending excitation was also investigated. To this end, the Gly-Alg-Glycerol film sensor was attached to an elastic polydimethylsiloxane (PDMS) substrate, and was cyclically bended by a vibration push rod (Figure 3F), meanwhile the output voltage was recorded. We define the bending angle θ = tan^−1^ (h/L), and the sensor was bended at 5°, 10° and 15° with a frequency of 0.8 Hz. The output voltage increased from 0.08 to 0.35 V when the bending angle increased from 5° to 15°, revealing that larger deformation leads to stronger piezoelectric response (Figure 3G). Furthermore, the influence of the frequency of the bending deformation on the piezoelectric response of the film was studied, and the result was shown in Figure 3H, from which one can see, the output voltage increased with the increase of the bending frequency ranging from 0.2–1 Hz. The high strain rate induced by the high bending frequency may be responsible for this phenomenon according to the equation of $V=dEA_{E}\dot{\varepsilon }R,\;$where *V* is the output voltage, *d* is the piezoelectric charge constant, *E* is the Young’s modulus, *A*_E_ is the effective area, $\dot{\varepsilon }$ is the applied strain rate and *R* is the inner resistance of the piezoelectric material [46, 47].

Taken together, the Gly-Alg-Glycerol film developed here exhibited excellent piezoelectric performance and flexibility, which is superior to most reported piezoelectric biomaterials (Supplementary Table S1), thus holding great potential for application in the fields of flexible bioelectronics.

### Sensing applications of Gly-Alg-Glycerol film

**Figure 4.**
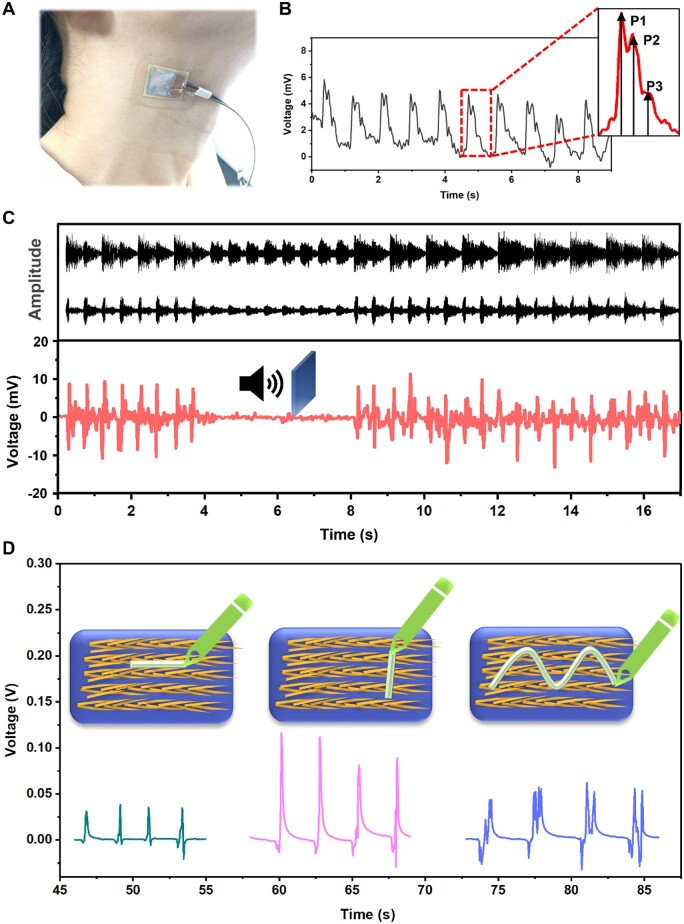
*In vitro* sensing performance of Gly-Alg-Glycerol film sensor. (**A**) Digital image of the Gly-Alg-Glycerol film sensor attached to human carotid artery. (**B**) Real-time output voltage of the sensor in response to carotid pulsation. The enlarged view shows the incident wave (P1), the tidal wave (P2) and the diastolic wave (P3) in one pulse cycle. (**C**) Output voltage waveform of the piezoelectric sensor in response to the sound wave signal from a speaker. (**D**) Output voltage waveform of the piezoelectric sensor in response to shear stress applied from different directions.

To demonstrate the functionality of Gly-Alg-Glycerol piezoelectric films in real-time health monitoring, we used the Gly-Alg-Glycerol piezoelectric sensor to detect subtle pulse signal of human carotid artery (Figure 4A). As shown in Figure 4B, the Gly-Alg-Glycerol piezoelectric sensor could sense the subtle pulse signal precisely and generated a stable output voltage in real time. Moreover, the sensor successfully detected a heart rate of 75 min^−1^ of the person in a calm state and the output voltage exhibited a specific waveform that reflects the characteristic of normal cardiac function, in which the incident wave (P1), the tidal wave (P2) and the diastolic wave (P3) could be distinguished clearly. In addition to subtle pulse signal, the Gly-Alg-Glycerol piezoelectric sensor could also detect sound wave signal. We placed the sensor in front of a speaker. When the speaker was turn on, one can see the sensor could generate time-dependent piezoelectric output voltage in response to the sound wave (Figure 4C). Moreover, the position and intensity of the voltage waveform precisely matched with the waveform of the sound source. This result indicates that the Gly-Alg-Glycerol piezoelectric film developed here has potential for application in artificial cochlea [48].

It is worth noting that the flexible Gly-Alg-Glycerol sensor could not only respond to vertical impulse force and bending deformation but also lateral shear stress. Interestingly, applying the shear stress from different directions resulted in dramatically different signal output. In other words, the piezoelectric Gly-Alg-Glycerol piezoelectric sensor was smart enough to know how the shear stress was applied. As shown in (Figure 4D), when a pen wrote on the sensor in the direction parallel to the β-Gly crystal orientation, the sensor generated a relatively weak output voltage. However, when the pen wrote on the sensor in the direction perpendicular to the β-Gly crystal orientation, the sensor generated a much higher output voltage. Furthermore, when the pen moved in a zigzag pattern across the sensor, a new output signal with special waveform was observed. We believe applying shear stress from different directions will cause different polarization behavior of β-Gly crystals, thereby resulting in different signal output. Due to its unique ability to recognize different writing activities, the Gly-Alg-Glycerol piezoelectric film developed here may be used in writing electronic devices in the future.

### Biocompatibility and *in vivo* sensing of Gly-Alg-Glycerol film

**Figure 5.**
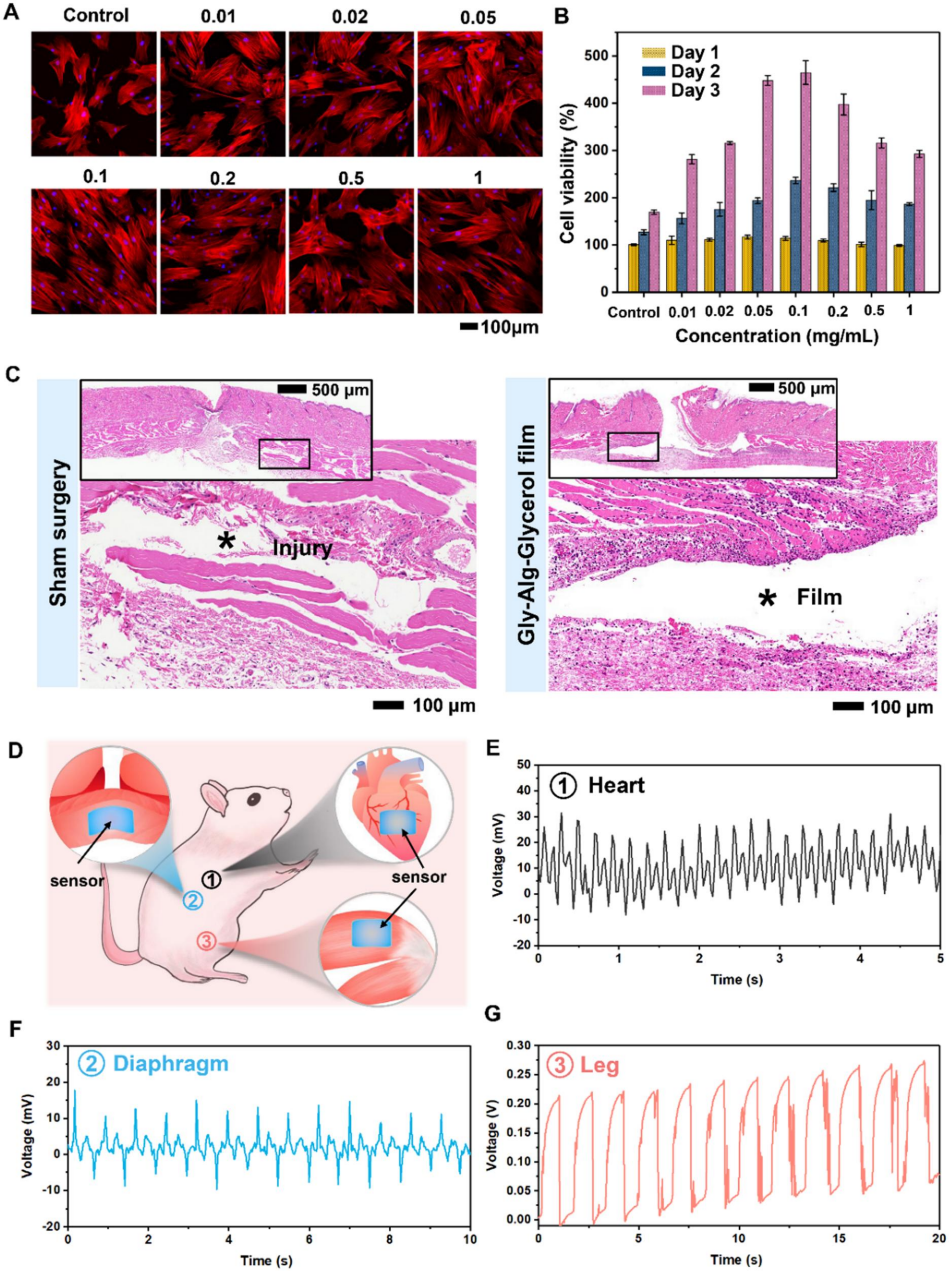
Biocompatibility, degradability and *in vivo* sensing performance of Gly-Alg-Glycerol film. (**A**) Fluorescence microscopy images showing the morphology of MSCs cultured for 2 days in culture medium containing different concentrations (0, 0.01, 0.02, 0.05, 0.1, 0.2, 0.5 and 1 mg/ml) of Gly-Alg-Glycerol. Red indicates the cytoskeleton, and blue indicates the nucleus. (**B**) Cell viability of MSCs cultured in culture medium containing different concentrations (0, 0.01, 0.02, 0.05, 0.1, 0.2, 0.5 and 1 mg/ml) of Gly-Alg-Glycerol. (**C**) H&E stained images of dorsal subcutaneous tissue implanted with no material and the Gly-Alg-Glycerol after 1 day of implantation. (**D**) Schematic illustration of the implantation sites of the Gly-Alg-Glycerol sensor in SD rat. (**E**) Real-time output voltage generated by the sensor attached to the outer wall of the heart of a SD rat. (**F**) Real-time output voltage generated by the sensor implanted into the base of the diaphragm of a SD rat. (**G**) Real-time output voltage generated by the sensor attached of the surface of the thigh muscle of a SD rat.

The biocompatibility of the Gly-Alg-Glycerol was assessed by observing the morphology and measuring the viability of rat mesenchymal stem cells (MSCs) cultured in a minimal essential medium (α-MEM) containing different amount of Gly-Alg-Glycerol. The morphology of MSCs cultured for 2 days was studied using fluorescence staining and microscopy. As shown in Figure 5A, all cells exhibited a spread morphology with elongated actin cytoskeleton. No obvious difference was observed among different groups. Live/dead staining revealed non-cytotoxicity of Gly-Alg-Glycerol film as MSCs cultured in the presence of Gly-Alg-Glycerol showed comparable viability to those cultured on tissue culture plates (control groups), and nearly no dead cells were observed (Supplementary Figure S10). A Cell Counting Kit-8 (CCK-8) assay was further performed to confirm the biocompatibility of the Gly-Alg-Glycerol. At day 1, the viability of MSCs cultured with different amounts of Gly-Alg-Glycerol was similar to that of the control group, and then showed a significant increase from day 1 to day 3. Interestingly, at day 2 and day 3, the viability of MSCs cultured with Gly-Alg-Glycerol became much higher than that of the control group, indicating the Gly-Alg-Glycerol could promote the proliferation of MSCs. According to the literature, this effect may be attributed to the presence of Gly [49–51]. In addition, the hemolysis ratio is also an important data that reflects the biocompatibility of materials. In our research, the hemolysis ratios of Gly-Alg-Glycerol were all less than 2% at different concentrations, which indicated that Gly-Alg-Glycerol film could be regarded as blood-compatible materials (Supplementary Figure S11).

The *in vivo* biocompatibility and biodegradability of the Gly-Alg-Glycerol films were evaluated by implanting them into the dorsal subcutaneous sites of SD rats. A sham surgery group that received the same operation with no implantation was used as a control. After 1 day of implantation, the Gly-Alg-Glycerol film completely disappeared due to its water-soluble nature. To accurately evaluate the degradation rate, the Gly-Alg-Glycerol film was monitored by immersing it in PBS solution at 37°C. As shown in Supplementary Figure S12, the Gly-Alg-Glycerol film dissolved gradually over time, and was hardly seen after 2 h, indicating its excellent degradability via dissolution. According to hematoxylin-eosin (H&E) staining, no obvious damage was observed, but mild inflammatory reaction in both groups (Figure 5C). Further histological staining at days 2, 4 and 7 showed gradual healing of the subcutaneous pocket (Supplementary Figure S13), indicating the good *in vivo* biocompatibility and biodegradability of the Gly-Alg-Glycerol film.

Based on the excellent biocompatibility of the Gly-Alg-Glycerol piezoelectric film, we then explored its application in *in vivo* sensing. It should be noted that the Gly-Alg-Glycerol films are water soluble. Therefore, we used biodegradable polylactic acid (PLA) and bioabsorbable beeswax membrane as a physical barrier to seal the sensor to prevent it from dissolution in physiological body fluid [52]. The stability of the encapsulated sensor was tested by immerging the sensor in PBS. As shown in Supplementary Figure S14, the PLA and beeswax membrane could protect the sensor from dissolution for 5 days. On the sixth day, the output voltage of the sensor decreased dramatically, indicating the leakage of the physical barrier. In order to investigate the *in vivo* sensing performance, we implanted the encapsulated sensor into different parts of SD rats including the outer wall of heart, the base of diaphragm, and the thigh muscle surface (Figure 5D and Supplementary Figure S15). Real-time monitoring of the dynamic pressure at the outer wall of heart can provide abundant information reflecting the structural and functional characteristics of heart, which is of great significance for early diagnosis and intervention of cardiovascular related diseases [53, 54]. The Gly-Alg-Glycerol piezoelectric sensor developed here was shown to be able to generate a rhythmic pulse voltage signal in response to cardiac pulsation (Figure 5E). According to the output signal, the heart rate of the rat in a coma state was deduced to be 300 beats per minute, which agrees with previous literature [54]. We then implanted the same sensor into the base of a rat’s diaphragm in the abdomen. The real-time and continuous output voltage signal generated by the sensor was synchronized with the rat’s respiration (Figure 5F), revealing the respiratory frequency of the rat was about 78 breaths per minute. The distinguishability of the output signal is much superior to that of the poly-l-lactic acid (PLLA) pressure sensor reported in previous study [12]. Last but not least, the sensor was also able to detect the deformation of muscle during exercise. As shown in Figure 5G, the sensor attached on the surface of the thigh muscle generated a stable output voltage of ∼200 mV when the rat’s leg was stretched and released cyclically at a frequency of ∼0.6 Hz. These *in vivo* tests indicate that the Gly-Alg-Glycerol piezoelectric film developed here holds great potential for *in vivo* sensing.

## Conclusion

In this work, a flexible, biocompatible and biodegradable piezoelectric film with excellent *in vitro* and *in vivo* sensing performance was developed by a simple solvent-casting method. The piezoelectric film was composed of a single, monolithic β-Gly spherulite embedded in Alg-Glycerol matrix, with a d_33_ piezoelectric constant up to 7.2 ± 0.1 pC/N. Unlike conventional rigid piezoelectric materials, the piezoelectric film developed here was very flexible, enabling complex shape, e.g. origami pigeon, and repeated bending and folding deformation without fracture. Moreover, the piezoelectric film exhibited very good piezoelectric sensitivity and stability, which could generate stable output voltages in response to different pressure and bending deformation. *In vitro*, the Gly-Alg-Glycerol promoted the proliferation of MSCs and the Gly-Alg-Glycerol piezoelectric sensor could precisely sense the subtle pulse signal of human carotid artery, sound wave signal, as well as shear stress applied from different directions. *In vivo*, the Gly-Alg-Glycerol film was fully degradable, causing no strong immune response, and the encapsulated sensor showed outstanding performance in monitoring the dynamic signals of different tissues/organs including the outer wall of heart, the base of diaphragm, and the thigh muscle surface. Taken together, the Gly-Alg-Glycerol piezoelectric film developed here forms an appealing alternative to current rigid piezoelectric materials for biomedical sensing applications.

## Supplementary Material

rbae047_Supplementary_Data
