# Supplementary material for: Glycine/alginate-based piezoelectric film consisting of a single, monolithic β-glycine spherulite towards flexible and biodegradable force sensor
Source: Regen Biomater. 2024 May 11;11:rbae047. doi: 10.1093/rb/rbae047 (PMC11187499; doi:10.1093/rb/rbae047)
Supplement: rbae047_Supplementary_Data [file rbae047_supplementary_data.zip › Supporting Information.docx]

Supporting Information

### Glycine/alginate-based piezoelectric film consisting of a single, monolithic β-glycine spherulite towards flexible and biodegradable force sensor

Qiaoxia Lin ^1, 2, 4, ϯ^, Yonggang Zhang ^1, ϯ^, Luhua Chen ^3^, Haoyue Zhang ^1, 2, 4^, Chuanfeng An ^1^, Chengze Li ^1^, Qifan Wang ^1^, Jinhui Song ^3^, Wei He ^2, 4^, Huanan Wang ^1, 2, *^

^1^ MOE Key Laboratory of Bio-Intelligent Manufacturing, Dalian Key Laboratory of Artificial Organ and Regenerative Medicine, School of Bioengineering, Dalian University of Technology, Dalian 116024, Liaoning, P. R. China

^2^ State Key Laboratory of Fine Chemicals, Frontiers Science Center for Smart Materials Oriented Chemical Engineering, Dalian University of Technology, Dalian 116024, Liaoning, P. R. China

^3^ School of Mechanical Engineering, Dalian University of Technology, Dalian 116024, Liaoning, P. R. China

^4^ School of Chemical Engineering, Dalian University of Technology, Dalian 116024, Liaoning, P. R. China

^ϯ^ These authors contributed equally to this work.

^∗^ Corresponding author: Huanan Wang ([huananwang@dlut.edu.cn](mailto:huananwang@dlut.edu.cn))


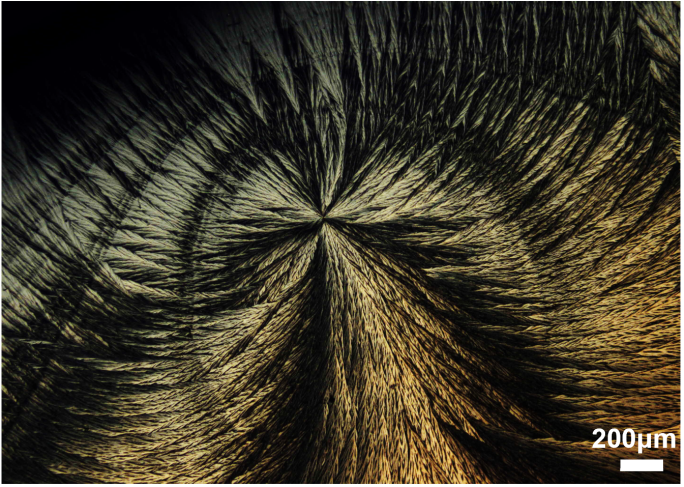


**Figure S1**. POM images of the nucleation center of the β-glycine spherulite in the Gly-Alg-Glycerol film.


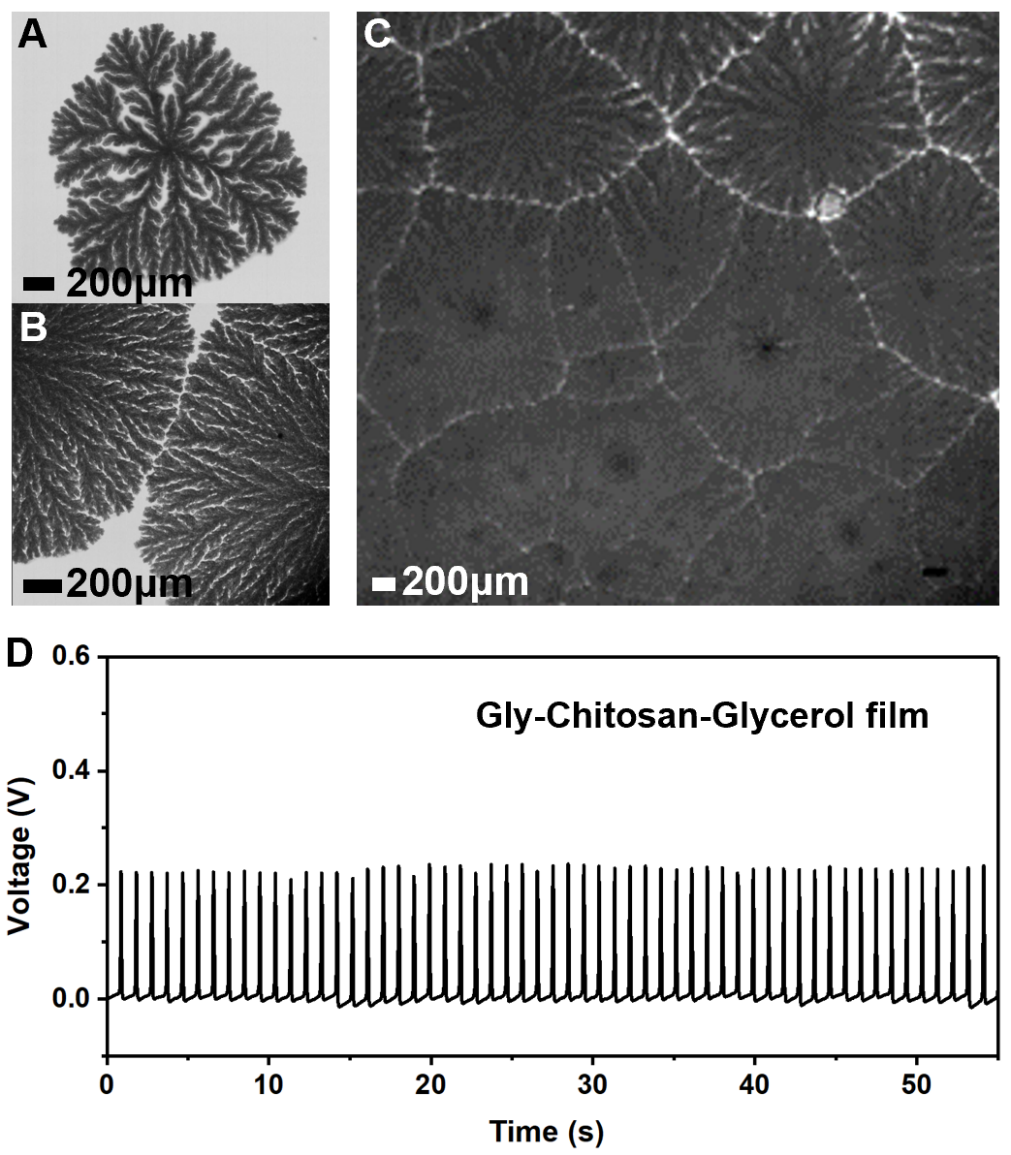


**Figure S2**. POM images of the Gly-Chitosan-Glycerol film showing the spherulite nucleus (A), grain boundary (B) and multiple spherulite film (C). Output voltage generated by the Gly-Chitosan-Glycerol film under a cyclic impulse force of 40 N with a frequency of 1 Hz. The Gly-Chitosan-Glycerol film was fabricated using the same method. Briefly, 40ml of a homogeneous solution containing 0.4 g of chitosan, 0.4 g of Gly, and 0.4 g of glycerol was casted into a polystyrene petri dish with a diameter of 115 mm and dried for 1week.


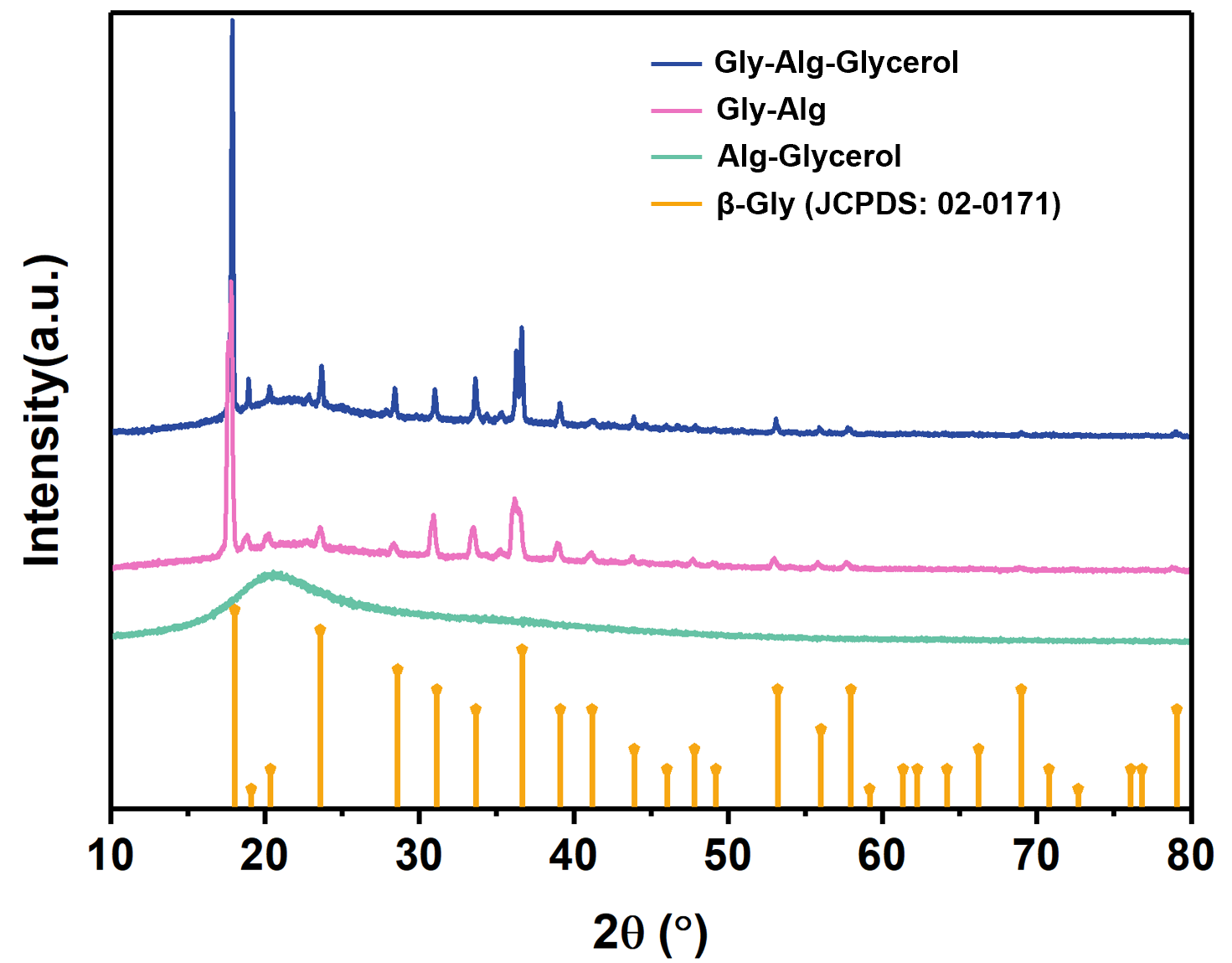


**Figure S3**. XRD spectra of the Gly-Alg-Glycerol film with 1% glycerol and a Gly/Alg ratio of 1:1, Gly-Alg film and Alg-Glycerol film prepared by the same method.


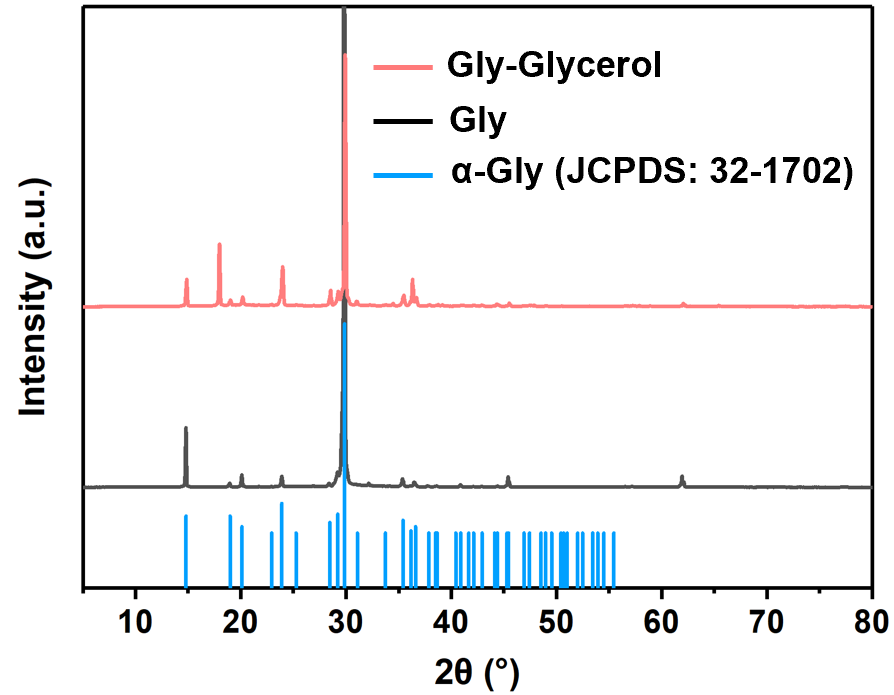


**Figure S4**. The XRD spectra of pure Gly and Gly-Glycerol, which were formed through solvent-casting using a 1% Gly aqueous solution and a mixed solution comprising 1% Gly and 1% Glycerol, respectively.


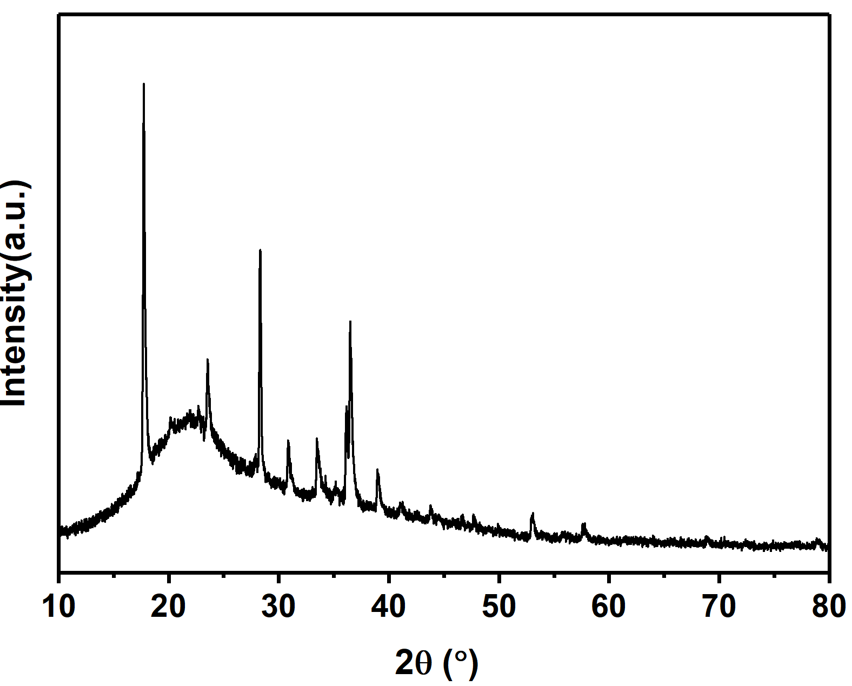


**Figure S5**. XRD spectrum of Gly-Alg-Glycerol film after six months of storage.


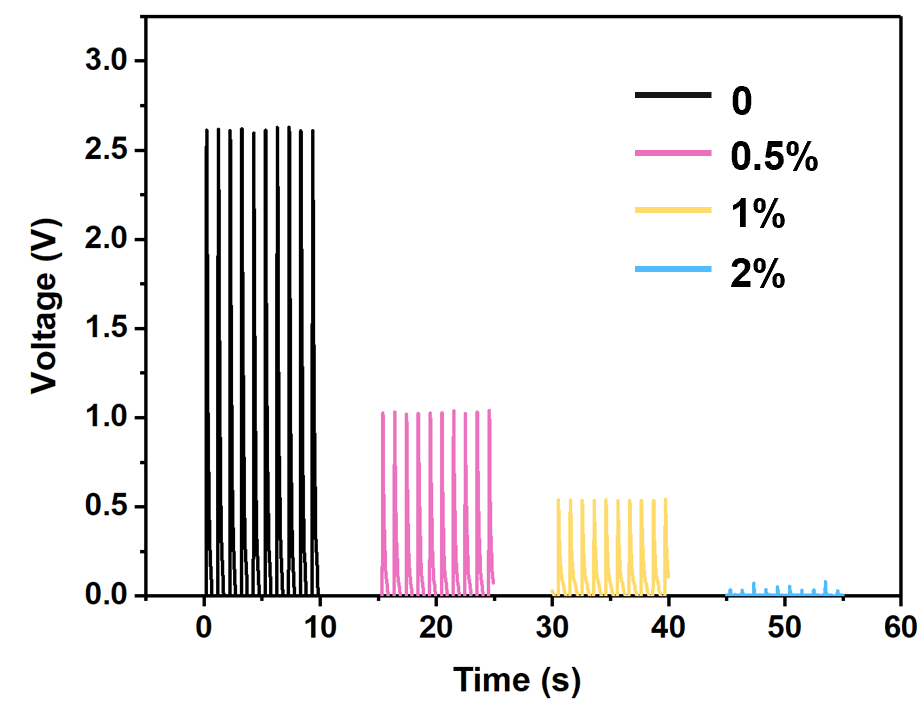


**Figure S6**. Piezoelectric output voltage of Gly-Alg-Glycerol films (50 μm in thickness) with a Gly/Alg ratio of 1:1 and different amount of glycerol under a cyclic impulse force of 40 N at a frequency of 1 Hz over an effective area of 176.7 mm^2^.


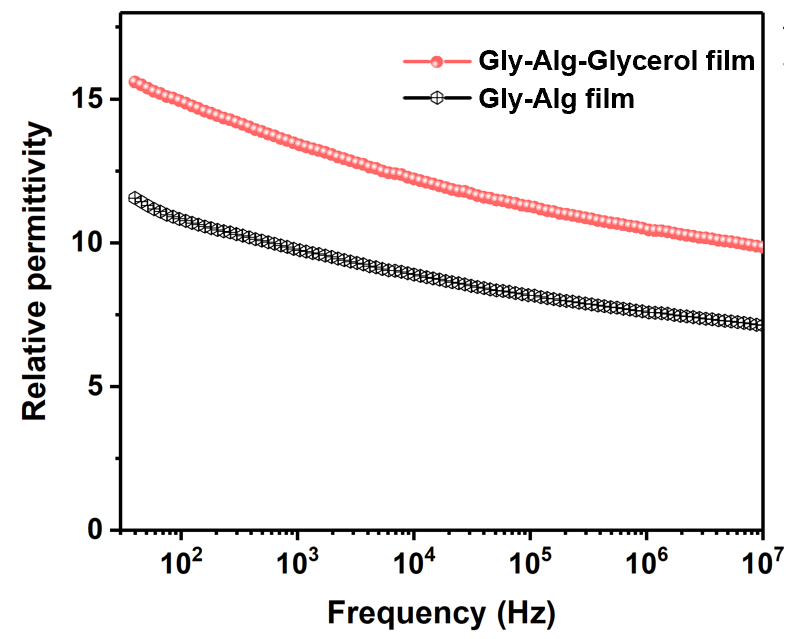


**Figure S7**. Relative permittivity of Gly-Alg-Glycerol film (1% glycerol) and Gly-Alg film (0% glycerol) with a Gly/Alg ratio of 1:1.


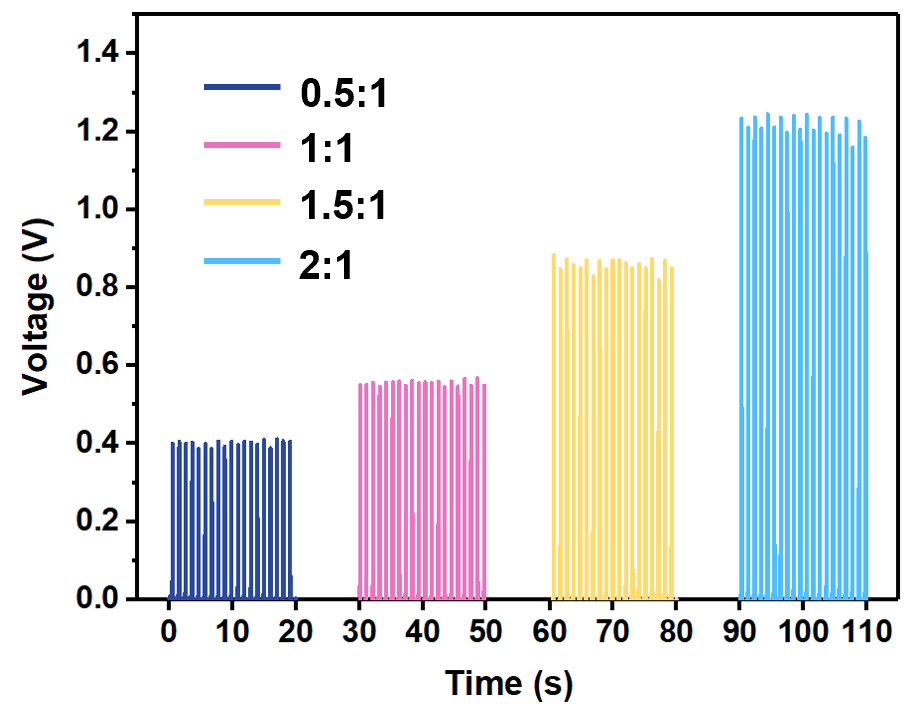


**Figure S8**. Piezoelectric voltage output of Gly-Alg-Glycerol films (50 μm in thickness) with 1% glycerol and different Gly/Alg mass ratios under a cyclic impulse force of 40 N at a frequency of 1 Hz over an effective area of 176.7 mm^2^.


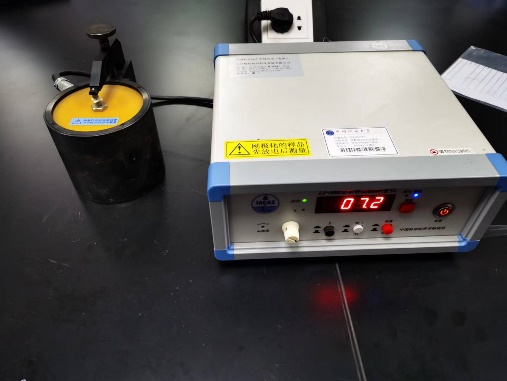


**Figure S9**. Measuring the d_33_ piezoelectric constant of a Gly-Alg-Glycerol film with 1% glycerol and a Gly/Alg ratio of 1:1 by using a commercial d_33_ meter.


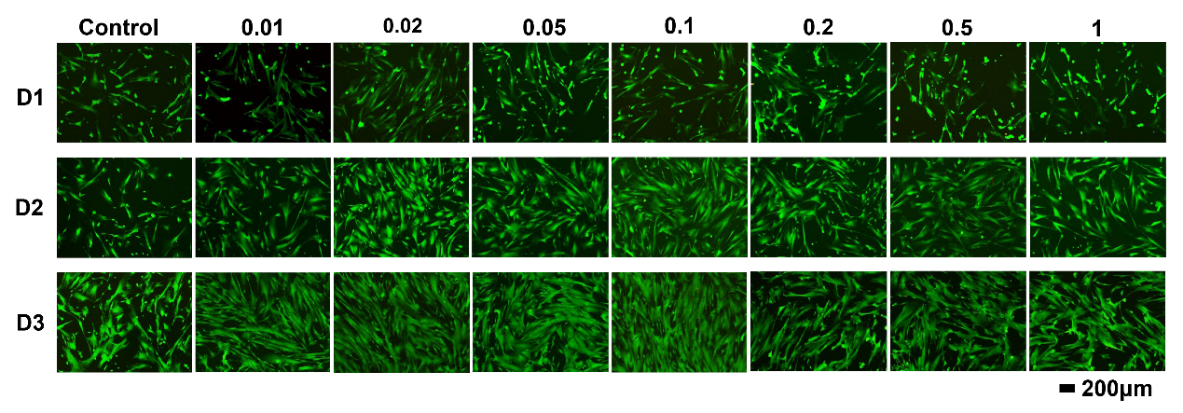


**Figure S10**. Live/dead staining of MSCs cells cultured for 3 days in culture medium solutions containing different concentrations (0, 0.01, 0.02, 0.05, 0.1, 0.2, 0.5 and 1 mg/ml) of Gly-Alg-Glycerol. Green represents live MSCs, red represents dead MSCs.


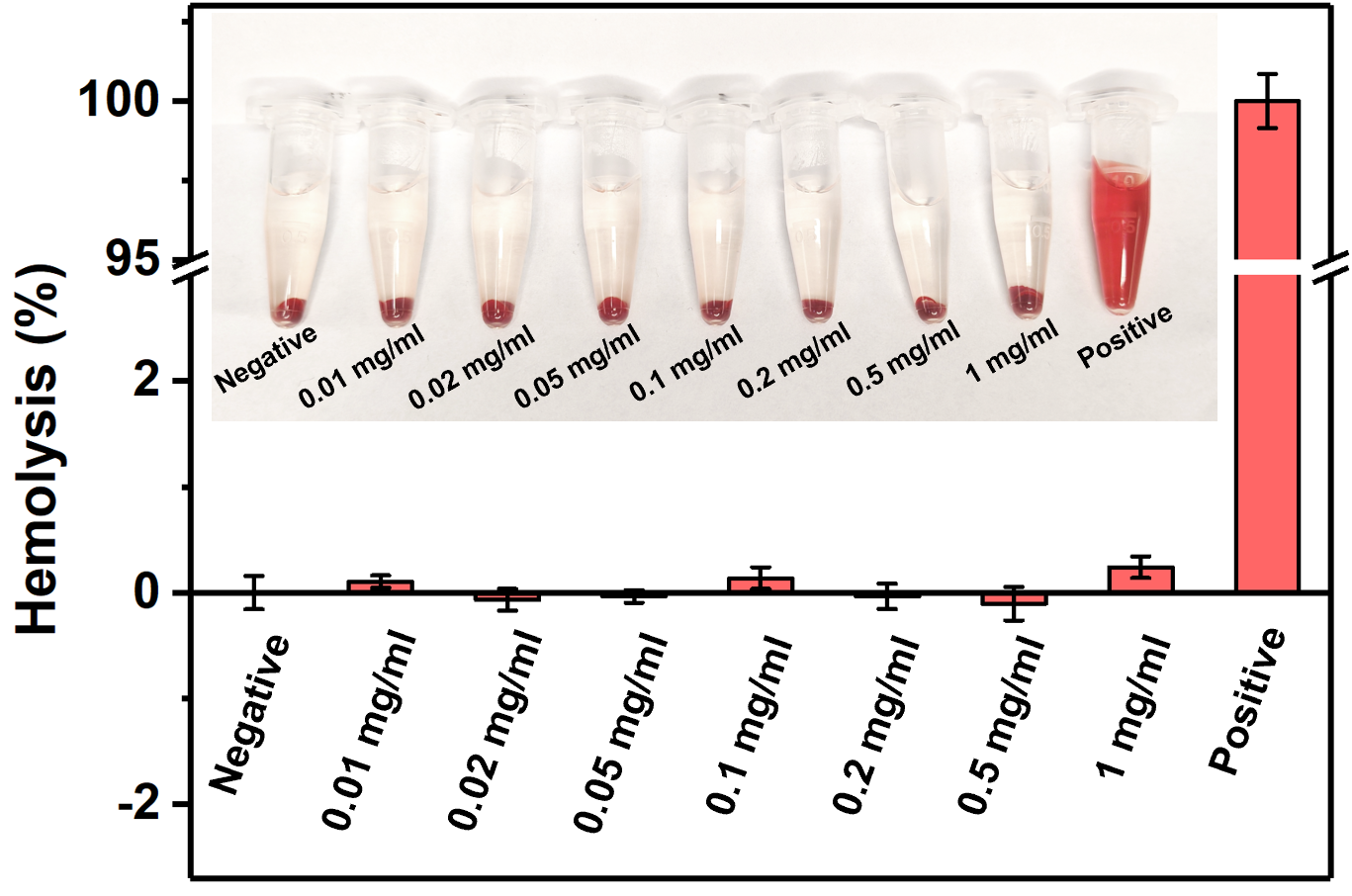


**Figure S11**. Hemocompatibility of Gly-Alg-Glycerol film. Standard saline solution was used as a negative control and distilled water as a positive control, respectively.


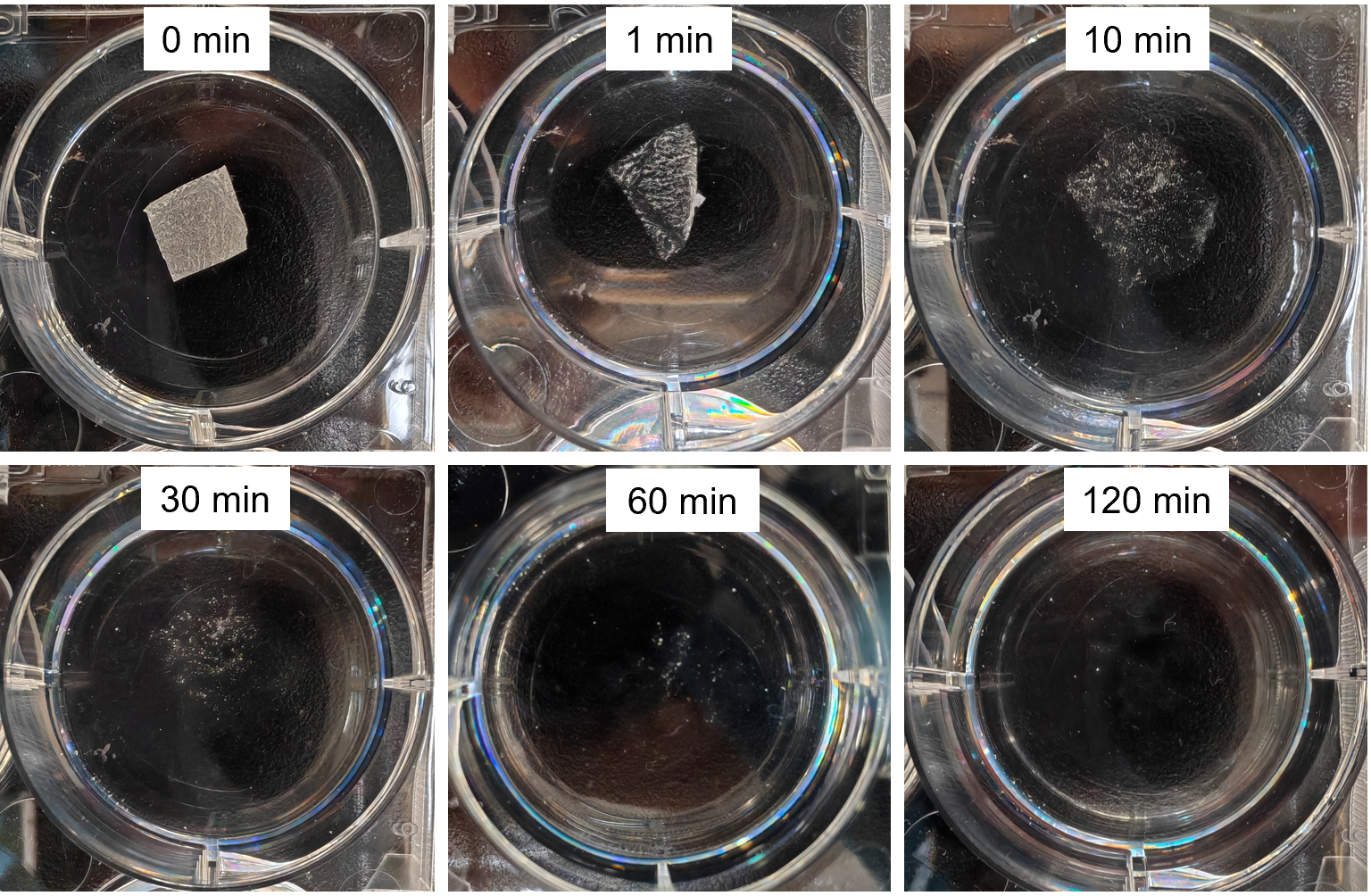


**Figure S12**. Optical images showing the solubility of Gly-Alg-Glycerol film in the PBS solution with time.


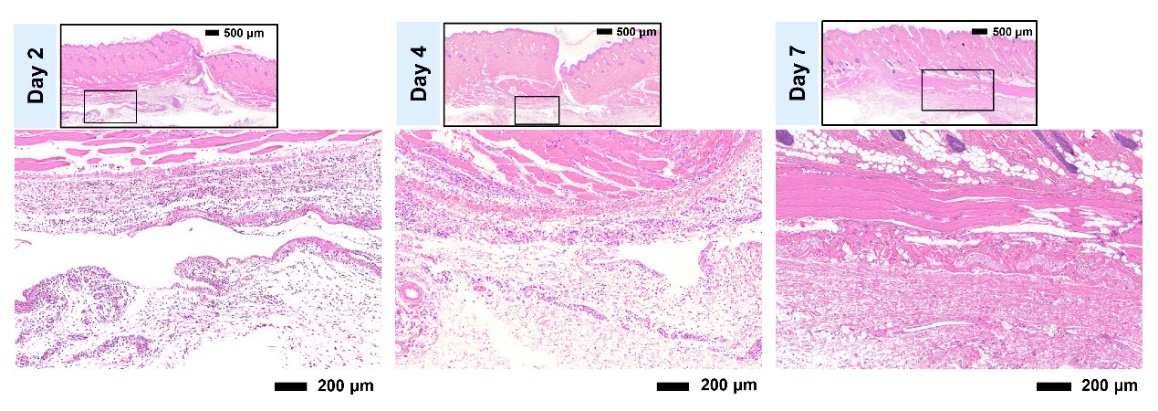


**Figure S13**. Representative H&E stained images after 2, 4 and 7 days of subcutaneous implantation.


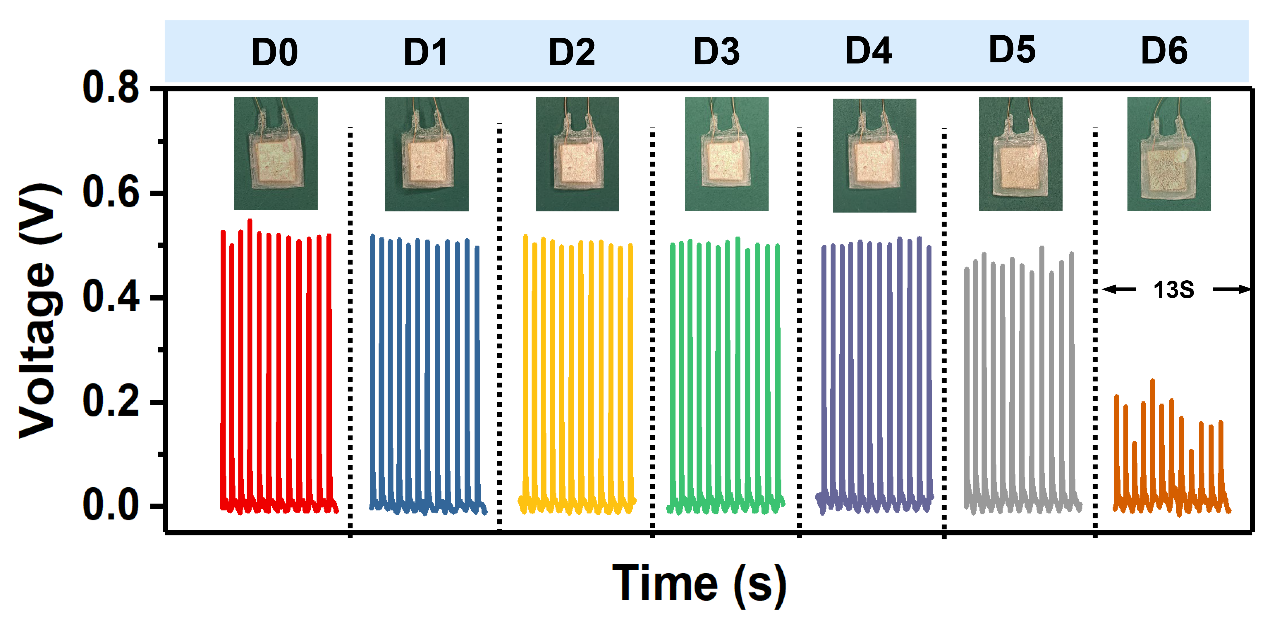


**Figure S14**. Time-dependent piezoelectric output voltages under a cyclic impulse force of 40 N at a frequency of 1 Hz over an effective area of 176.7 mm^2^ and digital photographs of the encapsulated Gly-Alg-Glycerol sensor during 6 days’ stability test in PBS buffer solution.


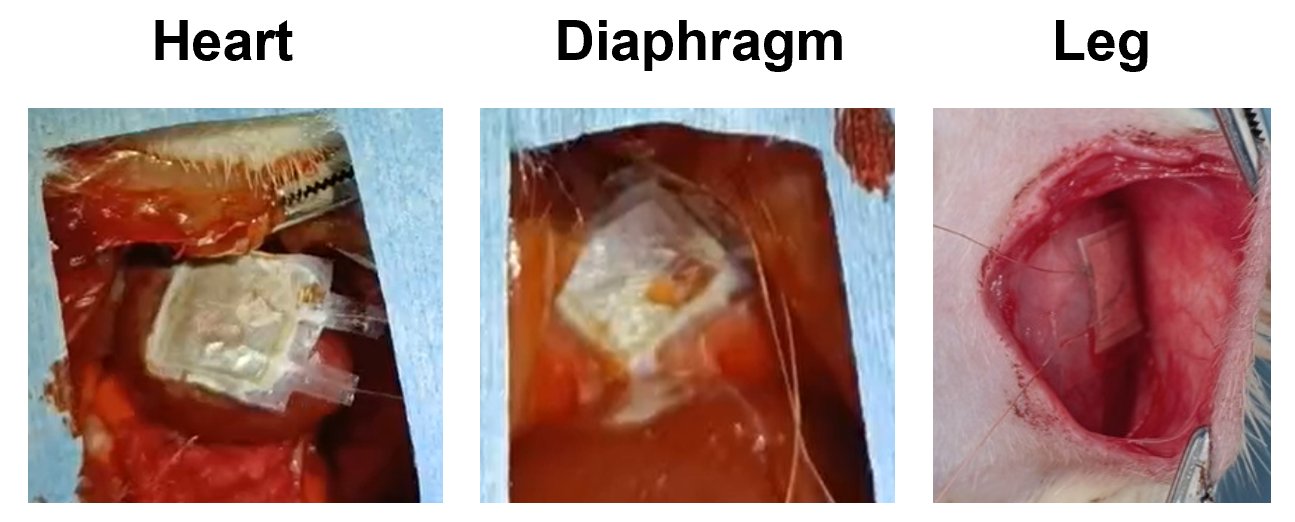


**Figure S15**. Digital photographs of the Gly-Alg-Glycerol sensors in the different areas of SD rats.

**Table S1**. Comparison of the related performances of this work with the previous work.

| Materials | | d33  (pC/N or pm/V) | Flexibility | Biodegradability | Biocompatibility | Ref. |
| --- | --- | --- | --- | --- | --- | --- |
| Biomaterials | Collagen | 0.89 | / | Biodegradable | Biocompatible | [1, 2] |
|  | Chitin | 4 | Bendable | Biodegradable | / | [3] |
|  | Virus | 7.8 | / | Biodegradable | / | [4, 5] |
|  | Cellulose | 5.7 | Bendable | / | / | [6] |
|  | DL-alanine | 5.5 | Stretchable (40%) | Biodegradable | Biocompatible | [7] |
|  | Isoleucine | 1.2 | / | Biodegradable | Biocompatible | [8] |
|  | Gly-PVA | 5.3 | Stretchable (~0.2%) | Biodegradable | Biocompatible | [9] |
|  | Gly-CS | / | Bendable | Biodegradable | / | [10] |
|  | **This work** | **7.2** | **Bendable, stretchable (40%), foldable** | **Biodegradable** | **Biocompatible** |  |
| Synthetic polymer | PLLA | ~5-25 | Flexible | Biodegradable | Biocompatible | [11, 12] |
|  | PVDF | -33 | Flexible | Nondegradable | Potential toxic | [13] |
| Inorganic materials | PZT-5H | 593 | Non-flexible | Nondegradable | Potential toxic | [13] |
|  | BaTiO_3_ | 275 | Non-flexible | Nondegradable | Biocompatible | [14, 15] |

**References：**

1. Denning D, Kilpatrick JI, Fukada E, Zhang N, Habelitz S, Fertala A, Gilchrist MD, Zhang Y, Tofail SAM, Rodriguez BJ. Piezoelectric Tensor of Collagen Fibrils Determined at the Nanoscale. *Acs Biomater Sci Eng* **2017**;3:929-935.

2. Laurent GJ. Dynamic State of Collagen - Pathways of Collagen Degradation Invivo and Their Possible Role in Regulation of Collagen Mass. *Am J Physiol* **1987**;252:C1-C9.

3. Kim K, Ha M, Choi B, Joo SH, Kang HS, Park JH, Gu B, Park C, Park C, Kim J, Kwak SK, Ko H, Jin J, Kang SJ. Biodegradable, Electro-active Chitin Nanofiber Films for Flexible Piezoelectric Transducers. *Nano Energy* **2018**;48:275-283.

4. Tothova Lu, Babickova J, Celec P. Phage Survival: The Biodegradability of M13 Phage Display Library in Vitro. *Biotechnol Appl Bioc* **2012**;59:490-494.

5. Lee BY, Zhang J, Zueger C, Chung W-J, Yoo SY, Wang E, Meyer J, Ramesh R, Lee S-W. Virus-based Piezoelectric Energy Generation. *Nat Nanotechnol* **2012**;7:351-356.

6. Rajala S, Siponkoski T, Sarlin E, Mettanen M, Vuoriluoto M, Pammo A, Juuti J, Rojas OJ, Franssila S, Tuukkanen S. Cellulose Nanofibril Film as a Piezoelectric Sensor Material. *Acs Appl Mater Inter* **2016**;8:15607-15614.

7. Li J, Carlos C, Zhou H, Sui J, Wang Y, Silva-Pedraza Z, Yang F, Dong Y, Zhang Z, Hacker TA, Liu B, Mao Y, Wang X. Stretchable piezoelectric biocrystal thin films. *Nat Commun* **2023**;14.

8. Cheng Y, Xu J, Li L, Cai P, Li Y, Jiang Q, Wang W, Cao Y, Xue B. Boosting the Piezoelectric Sensitivity of Amino Acid Crystals by Mechanical Annealing for the Engineering of Fully Degradable Force Sensors. *Adv Sci* **2023**;10:2207269.

9. Yang F, Li J, Long Y, Zhang Z, Wang L, Sui J, Dong Y, Wang Y, Taylor R, Ni D, Cai W, Wang P, Hacker T, Wang X. Wafer-scale Heterostructured Piezoelectric Bio-organic Thin Films. *Science* **2021**;373:337-342.

10. Hosseini ES, Manjakkal L, Shakthivel D, Dahiya R. Glycine-Chitosan-Based Flexible Biodegradable Piezoelectric Pressure Sensor. *Acs Appl Mater Inter* **2020**;12:9008-9016.

11. Tai YY, Yang S, Yu S, Banerjee A, Myung NV, Nam J. Modulation of piezoelectric properties in electrospun PLLA nanofibers for application-specific self-powered stem cell culture platforms. *Nano Energy* **2021**;89.

12. Curry EJ, Ke K, Chorsi MT, Wrobel KS, Miller AN, III, Patel A, Kim I, Feng J, Yue L, Wu Q, Kuo C-L, Lo KWH, Laurencin CT, Ilies H, Purohit PK, Nguyen TD. Biodegradable Piezoelectric Force Sensor. *P Natl Acad Sci Usa* **2018**;115:909-914.

13. Chorsi MT, Curry EJ, Chorsi HT, Das R, Baroody J, Purohit PK, Ilies H, Nguyen TD. Piezoelectric Biomaterials for Sensors and Actuators. *Adv Mater* **2019**;31:1802084.

14. Du Q, Zhang C, Liu C-L, Zhou W, Zeng F, Li K, Zou K, Fan G, Jiang S, Fu Q, Wu J-M, Zhang G. Highly <001>-textured BaTiO3 ceramics with high piezoelectric performance prepared by vat photopolymerization. *Addit Manuf* **2023**;66.

15. Wang X, Dai X, Chen Y. Sonopiezoelectric Nanomedicine and Materdicine. *Small* **2023**;19.
